# Body composition and lung cancer-associated cachexia in TRACERx

**DOI:** 10.1038/s41591-023-02232-8

**Published:** 2023-04-12

**Authors:** Othman Al-Sawaf, Jakob Weiss, Marcin Skrzypski, Jie Min Lam, Takahiro Karasaki, Francisco Zambrana, Andrew C Kidd, Alexander Frankell, Thomas B. K. Watkins, Carlos Martinez-Ruiz, Mateo Sokac, Susie Collins, Selvaraju Veeriah, Neil Magno, Cristina Naceur-Lombardelli, Paulina Prymas, Antonia Toncheva, Nick Jayanth, Roberto Salgado, Christopher P. Bridge, David Christiani, Raymond Mak, Camden Bay, Michael Rosenthal, Naveed Sattar, Paul Welsh, Ying Liu, Norbert Perrimon, Karteek Poporui, Mirza Faisal Beg, Nicholas McGranahan, Allan Hackshaw, Danna M Breen, Stephen O’Rahilly, Nicolai J. Birkbak, Hugo Aerts, TRACERx Consortium, Mariam Jamal-Hanjani, Charles Swanton

**Affiliations:** 1 Cancer Research UK Lung Cancer Centre of Excellence, University College London Cancer Institute, London, UK; 2 Cancer Metastasis Laboratory, University College London Cancer Institute, London, UK; 3 Cancer Evolution and Genome Instability Laboratory, The Francis Crick Institute, London, UK; 4 Artificial Intelligence in Medicine Program, Brigham and Women's Hospital, Harvard Medical School, Boston, MA, USA; Cardiovascular Imaging Research Center, Massachusetts General Hospital, Harvard Medical School, Boston, MA, USA; 5 Department of Diagnostic and Interventional Radiology, University Freiburg, Germany; 6 Medical University of Gdańsk, Department of Oncology and Radiotherapy, 17 Smoluchowskiego St., 80-001 Gdańsk, Poland; 7 Infanta Sofía University Hospital, Madrid, Spain; 8 Institute of Infection, Immunity & Inflammation, University of Glasgow, Glasgow (Glasgow), United Kingdom; 9 Department of Clinical Medicine, Aarhus University, Aarhus, Denmark; 10 Department of Molecular Medicine, Aarhus University Hospital, Aarhus, Denmark; 11 Early Clinical Development, Pfizer UK Ltd, Cambridge, UK; 12 Cancer Research UK & University College London Cancer Trials Centre, London, UK; 13 Breast Cancer Translational Research Laboratory. Institut Jules Bordet; Department of Pathology/TCRU GZA Antwerp, Belgium; 14 MGH & BWH Center for Clinical Data Science, Boston, MA, USA; 15 Harvard Education and Research Center for Occupational Safety and Health, Boston, MA, USA; 16 Radiation Oncology Disease Center, Brigham and Women’s Hospital, Boston, MA, USA; 17 Department of Radiology, Brigham and Women’s Hospital, Boston, MA, USA; 18 School of Cardiovascular and Metabolic Health, University of Glasgow, Glasgow, United Kingdom; 19 Department of Genetics, Harvard Medical School, Boston, United States; 20 Howard Hughes Medical Institute, Harvard Medical School, Boston, United States; 21 Department of Computer Science, Memorial University of Newfoundland, St. John's, NL, Canada; 22 School of Engineering Science, Simon Fraser University, Burnaby, BC, Canada; 23 Internal Medicine Research Unit, Pfizer Inc, Cambridge, MA, USA; 24 Wellcome Trust-MRC Institute of Metabolic Science and NIHR Cambridge Biomedical Research Centre, University of Cambridge, Cambridge, UK; 25 Department of Medical Oncology, University College London Hospitals, London, UK

## Abstract

Cancer-associated cachexia (CAC) is a major determinant of morbidity and mortality in patients with non-small cell lung cancer (NSCLC). Key features of CAC include alterations in body composition and body weight. Here, we explore the association between body composition and body weight with survival and delineate possible biological processes and mediators that contribute to the development of CAC. Computed tomography-based (CT) body composition analysis of 651 patients in TRACERx suggested that patients with low skeletal muscle or adipose tissue area at the time of lung cancer diagnosis, represented by the bottom 20^th^ percentile, had significantly shorter lung cancer-specific survival (LCSS) and overall survival (OS). This finding was validated in 420 patients in the independent Boston Lung Cancer Study. In a longitudinal subset of 272 patients in TRACERx who experienced disease relapse, loss of adipose tissue, skeletal muscle, or body weight in the interval between diagnosis and relapse, was significantly associated with shorter LCSS and OS. Patients with one or more features encompassing loss of adipose or muscle tissue, or BMI-adjusted weight loss according to specific thresholds were classified as having developed CAC and were found to have distinct tumour genomic and transcriptomic profiles compared with patients who did not develop such features at relapse. Primary NSCLCs from patients in the CAC group were characterised by enrichment of inflammatory signalling and epithelial-mesenchymal transitional pathways, and differentially expressed genes upregulated in these tumours included cancer-testis antigen *MAGEA6* and matrix metalloproteinases, such as *ADAMTS3*. In an exploratory analysis of putative circulating cachexia mediators performed in a subset of 256 baseline and relapse plasma samples from TRACERx, proteomic analysis revealed a significant association between circulating GDF15 and loss of body weight, skeletal muscle, and adipose tissue at relapse, supporting the potential therapeutic relevance of targeting GDF15 in the management of CAC.

## Introduction

Measures of body composition that distinguish skeletal muscle (SKM), visceral adipose tissue (VAT), and subcutaneous adipose tissue (SAT) are associated with clinical outcomes in various diseases, including cancer ^1–4^. One extreme manifestation of altered body composition that remains poorly understood is cancer-associated cachexia (CAC); a paraneoplastic syndrome of involuntary skeletal muscle and/or adipose tissue loss, accompanied by dysregulation of the homeostatic mechanisms that govern protein and energy balance ^5,6^.

Retrospective analyses have linked body composition to outcomes across multiple solid tumour types, including breast, prostate and colorectal cancers ^7–9^. In non-small cell lung cancer (NSCLC), SKM wasting has been shown to be associated with cancer treatment toxicity and reduced overall survival (OS) ^10^. A meta-analysis of 13 NSCLC cohort studies demonstrated an association between low SKM mass and reduced OS, but not disease-specific survival^11^; whilst retrospective studies have similarly suggested an association between low SAT and low VAT with reduced OS ^12,13^, again, without a significant difference in cancer-specific survival. There remain key areas of uncertainty; not least the dynamics of body composition with time, and the underlying molecular mediators of altered body composition in CAC.

Our study had three aims: (1) to systematically profile the body composition using standardised methods among patients diagnosed with early stage lung cancer in terms of SKM, VAT and SAT, and to explore associations between baseline measures and clinical outcomes, (2) to examine how these body composition measures change over time, and to derive an initial combined measure of CAC and observe how this is associated with clinical outcomes, and (3) to explore the corresponding tumour genomic, transcriptomic, and plasma proteomic landscape for potential molecular mechanisms and mediators of CAC.

We used established computed tomography (CT) imaging analysis methods ^14–16^ to assess body composition at lung cancer diagnosis and relapse, addressing the relevance of initial body composition metrics, and their dynamics at time of recurrence, to lung cancer outcomes and the cachectic phenotype. To investigate the interplay between tumour biology, body composition losses, and clinical outcomes, our analysis plan involved whole exome and transcriptomic analysis of diagnostic bulk tumour samples, in addition to plasma proteomics, to identify tumour-intrinsic factors, alongside potential circulating mediators, underpinning CAC.

**Figure 1:**
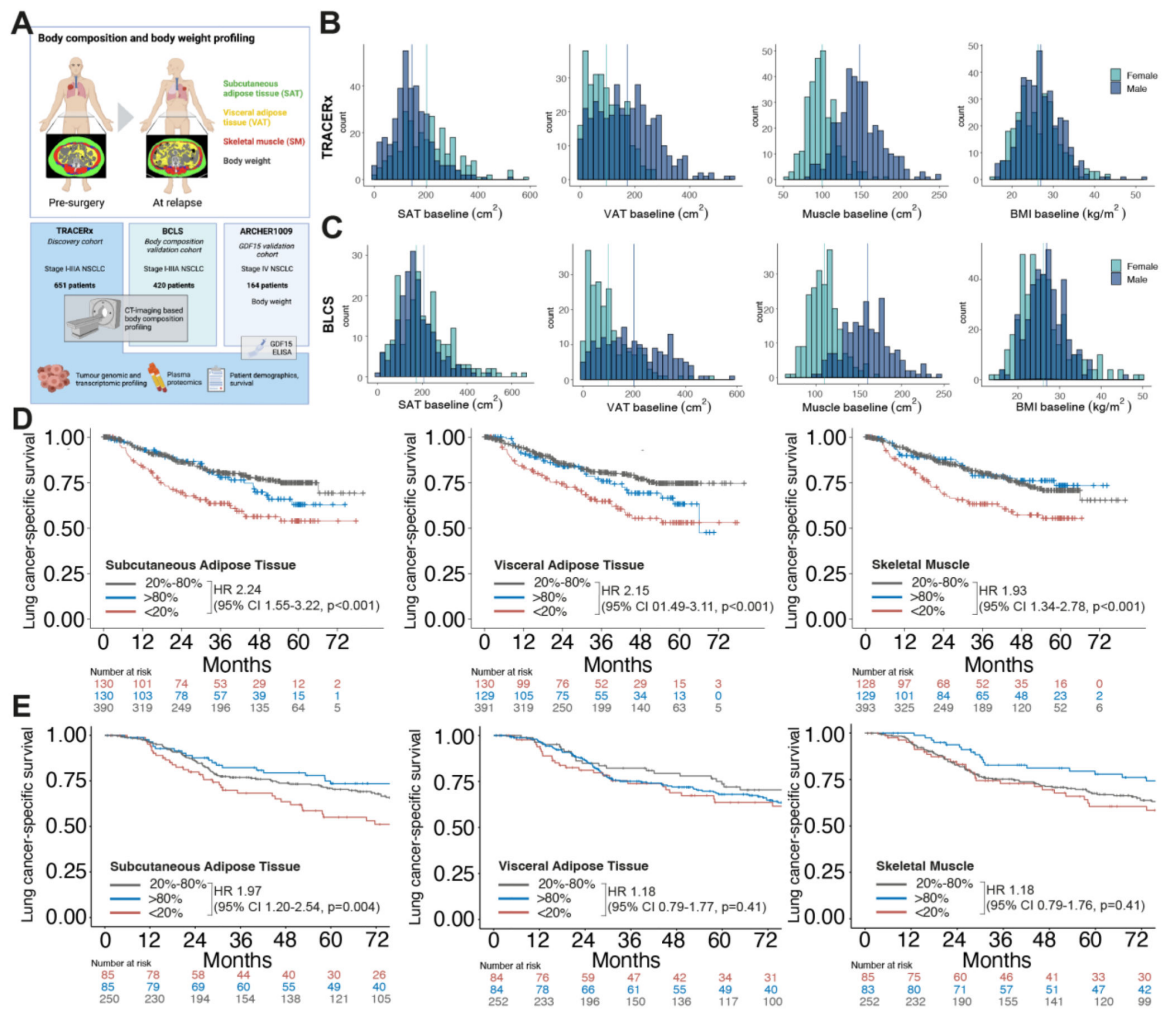
Body composition and cancer-specific survival in the TRACERx and BLCS studies. **A** Study outline of body composition and downstream analyses. **B** Distribution of body composition and body mass metrics according to sex at primary diagnosis in the TRACERx cohort (N=651) and **C** BLCS cohort (N=420); vertical lines indicate mean in female (green) and male (blue) patients. **D** Lung cancer-specific survival in TRACERx and **E** BLCS cohorts for body composition at primary diagnosis according to sex-specific bottom 20th percentile (red, <20%), mid 60th percentile (grey, 20%-80%) and upper 20th percentiles (blue, >80%) of subcutaneous adipose tissue (SAT), visceral adipose tissue (VAT) and skeletal muscle (SKM). Hazard ratios and 95% confidence intervals (CI) derived from univariate Cox regression analysis of the <20% group and the 20-80% groups.

We analysed CT imaging from two large independent cohorts of patients diagnosed with early stage operable NSCLC, treated with surgical resection, plus adjuvant therapy if indicated; the TRACERx (TRAcking non-small cell lung Cancer Evolution through therapy (Rx)) Lung study ^17,18^, our principal cohort of 651 patients, and the Boston Lung Cancer Study (BLCS)^19^, our validation cohort of 420 patients. Altogether, these encompassed 1071 diagnostic CT scans paired with detailed clinical annotation, including clinical outcomes. SAT, VAT and SKM areas at the third lumbar vertebrae level ^20^ were determined using a deep-learning based imaging analysis pipeline, establishing the baseline body composition database. In 272 patients from TRACERx, matched relapse body composition and/or body weight data were further evaluated to identify patients with changes indicative of CAC; primary bulk tumour samples from initial resection were analysed to infer the corresponding tumour genomic and transcriptomic landscape in relation to the CAC phenotype. Finally, we sought to establish the presence of circulating mediators associated with CAC by conducting plasma proteomic analysis on a subset of 128 patients from TRACERx (256 plasma samples collected at diagnosis and first relapse) to determine the differential protein expression in relation to our observed cachexia phenotype. By integrating longitudinal imaging, tumour, and blood analyses, we provide unique insights into the relationship between body composition and cachexia in NSCLC, characterising the tumour genome, transcriptome, and plasma proteome (Figure 1 A), establishing a platform for downstream validation and potential clinical translation.

## Results

### Low muscle and adipose tissue areas at diagnosis are associated with shorter survival

We profiled body composition, based on SAT, VAT and SKM tissue areas, of patients at the time of early stage NSCLC diagnosis to determine the prognostic associations of low SAT, VAT and SKM tissue areas on lung cancer specific-survival as the primary outcome measure for the body composition study. Body composition in 651 patients from the TRACERx study and 420 patients from the BLCS study was assessed at the time of cancer diagnosis. All patients had stage I-III disease and underwent primary resection. In the TRACERx cohort, 35% of the patients received adjuvant therapy (55% in the BLCS cohort) and 14% were current smokers (37% in the BLCS cohort), otherwise the baseline characteristics were similar in both cohorts (Table 1).

Body composition was measured by quantification of tissue area (cm^2^) at the level of the third lumbar vertebra (L3) on CT, or CT-PET, scan. SAT, VAT and SKM areas were quantified separately using deep-learning-based, automated pipelines (see Methods) ^21,22^. The distribution of body SAT, VAT, SKM and BMI was similar in the TRACERx and BCLS cohorts (Figure 1 B, C). In TRACERx and BLCS, male patients had higher VAT and SKM areas compared with female patients (TRACERx mean VAT 171.7 cm^2^ vs 95.1 cm^2^ [BLCS 198.5 cm^2^ vs 98.3 cm^2^], unpaired two-samples t-test p<0.001 and SKM 147.4 cm^2^ vs 99.5 cm^2^ [BLCS 160.2 cm^2^ vs 109.8 cm^2^], p<0.001), whereas female patients had higher SAT areas (TRACERx 201.2 cm^2^ vs 144.6 cm^2^, BLCS 206.5 cm^2^ vs 172.6 cm^2^; p<0.001) (Figure 1 B). There was a strong correlation between BMI and both SAT and VAT (Spearman’s correlation, r = 0.75 and 0.73, respectively), and a weaker correlation with SKM (r = 0.39) (Figure S1 A); similar correlations were observed in the BLCS cohort (Figure S1 B).

Patients in the TRACERx cohort were grouped into sex-adjusted bottom 20th, middle 20-80th and upper 20th percentiles, representing low, normal or high values of adipose and muscle tissue respectively (Figure 1D)., After adjusting for age, sex, BMI, smoking status, disease stage, histological subtype, ethnicity, and adjuvant therapy use, patients in the lowest 20^th^ percentile for SAT, VAT and SKM had significantly worse lung cancer-specific survival (LCSS) compared to patients in the middle 20-80^th^ percentile. The adjusted hazard ratios were 2.09 (95% CI 1.55-3.22, p<0.001), 1.73 (1.10-2.72, p=0.019) and 1.44 (0.95-2.19, p=0.088) for SAT, VAT and SKM, respectively (Table S1). Patients in the bottom 20th percentiles also had worse survival than those in the middle 20-80th percentile, with adjusted hazard ratios of 1.49 (1.02-2.16, p=0.037), 1.38 (0.95-2.01, p=0.093) and 1.28 (0.91-1.78, p=0.151) for SAT, VAT and SKM, respectively (Table S2).

These findings were confirmed in the BLCS cohort. Adjusted hazard ratios for LCSS of 1.97 (1.24-3.14, p=0.004), 1.57 (0.98-2.53, p=0.06), and 1.35 (0.86-2.12, p=0.19), for SAT, VAT and SKM, respectively (Figure 1 E, Table S3). For OS, the corresponding hazard ratios were 1.71 (95% CI 1.18-2.49, p=0.005), 1.75 (95% CI 1.20-2.54, p=0.003), and 1.41 (95% CI 1.00-2.01, p=0.05) (Table S4).

When patients were categorised into more extreme percentiles (lowest 10%, middle 10-90%, highest 10%), the associations between the lowest centiles and LCSS and OS remained similar or stronger (Table S5).

These independent cohorts show that patients with low SAT, VAT or SKM tissue at the time of NSCLC diagnosis have a shorter LCSS and OS compared with patients with normal values, indicating the potential prognostic value of baseline body composition.

### Longitudinal changes in body composition and body weight correlate with outcomes and identify the cachexia phenotype

Changes in body composition between diagnosis and relapse (median time to first recurrence 15.6 months, 95% CI 13.9-18.0) were examined in 188 patients in TRACERx who had a confirmed cancer recurrence and available abdominal CT image sections at these two timepoints (Figure S2). By using an automated, deep learning-based body composition measurement method (see Methods), and by manual quality control of all L3 annotations and segmentations, we ensured high precision of the longitudinal measurements.

**Figure 2:**
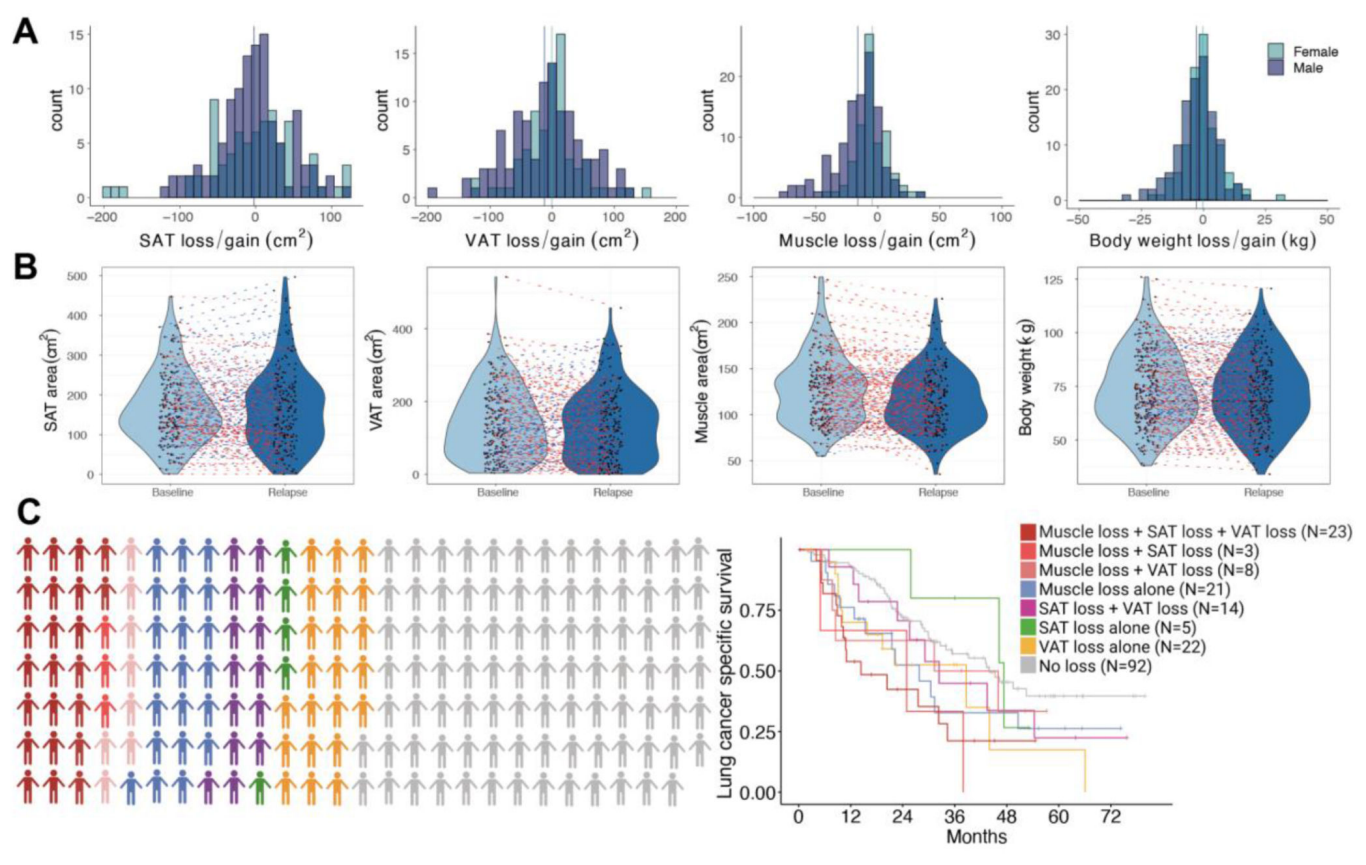
Survival outcomes according to changes in body composition between primary diagnosis and first relapse. **A** Distribution of losses and gains of body composition and body weight in the TRACERx cohort between primary diagnosis and first tumour recurrence according to sex (N=188). **B** Dynamics of body composition (N=188) and body weight (N=232) between primary diagnosis (“Baseline”) and first recurrence (“Relapse”), with red lines indicating decrease/loss, blue lines indicating increase/gain, and grey line indicating no changes. **C** Subgroups of patients according to (co-)presence of SAT loss (defined as ≥20% between baseline and relapse), VAT loss (≥20%) and SKM loss (≥10%). Kaplan-Meier analysis of lung cancer-specific survival according to labelled subgroups.

We used a range of cut-offs to define the absolute (cm^2^) change (i.e. gain and loss) in SKM, VAT, SAT and BMI-adjusted weight ^23^ (Figure 2 A, B). Regression analyses were then used to examine the association between these varying definitions of loss and LCSS (Methods, Figure S3).

A strong correlation was observed between body weight loss and VAT loss, and between VAT and SAT loss (Spearman’s r = 0.70 and 0.62, Figure S1 C). Likewise, grade 4 BMI-adjusted weight loss was associated with loss of SAT, VAT and SKM (Figure S1 D, p<0.0001).

A loss of ≥20% tissue area between diagnosis and relapse in SAT or VAT was associated with significantly worse LCSS (SAT: HR 1.56 [95% CI 1.02-2.38], p = 0.042; VAT: HR 2.34 [95% CI 1.38-3.99), p=0.0017) and worse OS (SAT: HR 1.59 [95% CI 1.079-2.36], p=0.019; VAT: HR 2.39 [95% CI 1.47-3.89], p<0.001) (Figure S4). While a loss of ≥10% skeletal muscle tissue area was associated with significantly worse LCSS and OS (HR 1.80 [95% CI 1.20-2.70], p=0.0047 and HR 1.85 [95% CI 1.27-2.69], p=0.0013). Grade 4 BMI-adjusted weight loss (see Methods) was significantly associated with worse LCSS and OS compared to patients with stable body weight (HR 5.637 [95% CI 3.19-9.95], p<0.0001 and HR 4.71 [95% CI 2.76-8.04], p<0.0001).

To explore whether distinct patterns of loss affecting specific body composition compartments were present, patients were grouped according to isolated or co-occurrence of SKM, VAT, and SAT losses for the above prognostic thresholds (Figure 2C). Amongst 96 patients in whom a loss occurred in any given compartment, 23/96 (24%) patients experienced losses across all three compartments, 25/96 (26%) patients experienced losses across two compartments and 48/96 (50%) patients experienced isolated loss in one compartment, that is., isolated loss of SKM (N=21), VAT (N=22) or SAT (N=5), suggesting possibly distinct clinical subtypes of CAC with different anatomical patterns of adipose and muscle tissue loss. Notably, patients with tissue loss across all three compartments had the shortest LCSS (HR: 2.6, 95% CI 1.39-4.87, p=0.003) (Figure 2C, Table S6).

Based on the visual inspection of each measure separately and the threshold for loss that appeared to be most strongly associated with LCSS and OS, we categorised patients into two groups: CAC (≥20% SAT and/or VAT loss, and/or ≥10% SKM loss, and/or grade 4 BMI-adjusted weight in the interval between diagnosis and relapse), and non-CAC (Figure S5). A higher proportion of males and squamous cell carcinoma histology subtype were seen in the CAC group compared to the non-CAC group (62.0% versus 54.2%, and 34.3% versus 25.8%), whereas smoking status and use of adjuvant therapy was similar between the two groups (Table S7). As expected, our defined CAC grouping (combining three measures of body mass loss and weight loss associated with poor outcome in this initial patient cohort) was associated with both LCSS and OS, after adjustment for sex, ethnicity, BMI, smoking status, histology, disease stage, and use of adjuvant therapy (HR 2.42 [95% CI 1.69-3.46] and HR 2.31 [95% CI 1.66- 3.20]) (Figure S5 C, Table S8).

Integrating significant changes in body composition and body weight identified patients who developed CAC between primary diagnosis and first relapse, and this annotation was used to conduct further downstream analyses in order to generate biological hypotheses relating to potential mediators of CAC.

### Primary tumour genomic and transcriptomic features in relation to body composition and body weight changes at first cancer relapse

In order to examine the presence of genomic and transcriptomic alterations potentially relevant to the development of CAC, we analysed tumour sequencing data in relation to the CAC and non-CAC groups. Primary NSCLC tumours from patients recruited into TRACERx were subjected to multi-region whole-exome and RNA sequencing.

**Figure 3:**
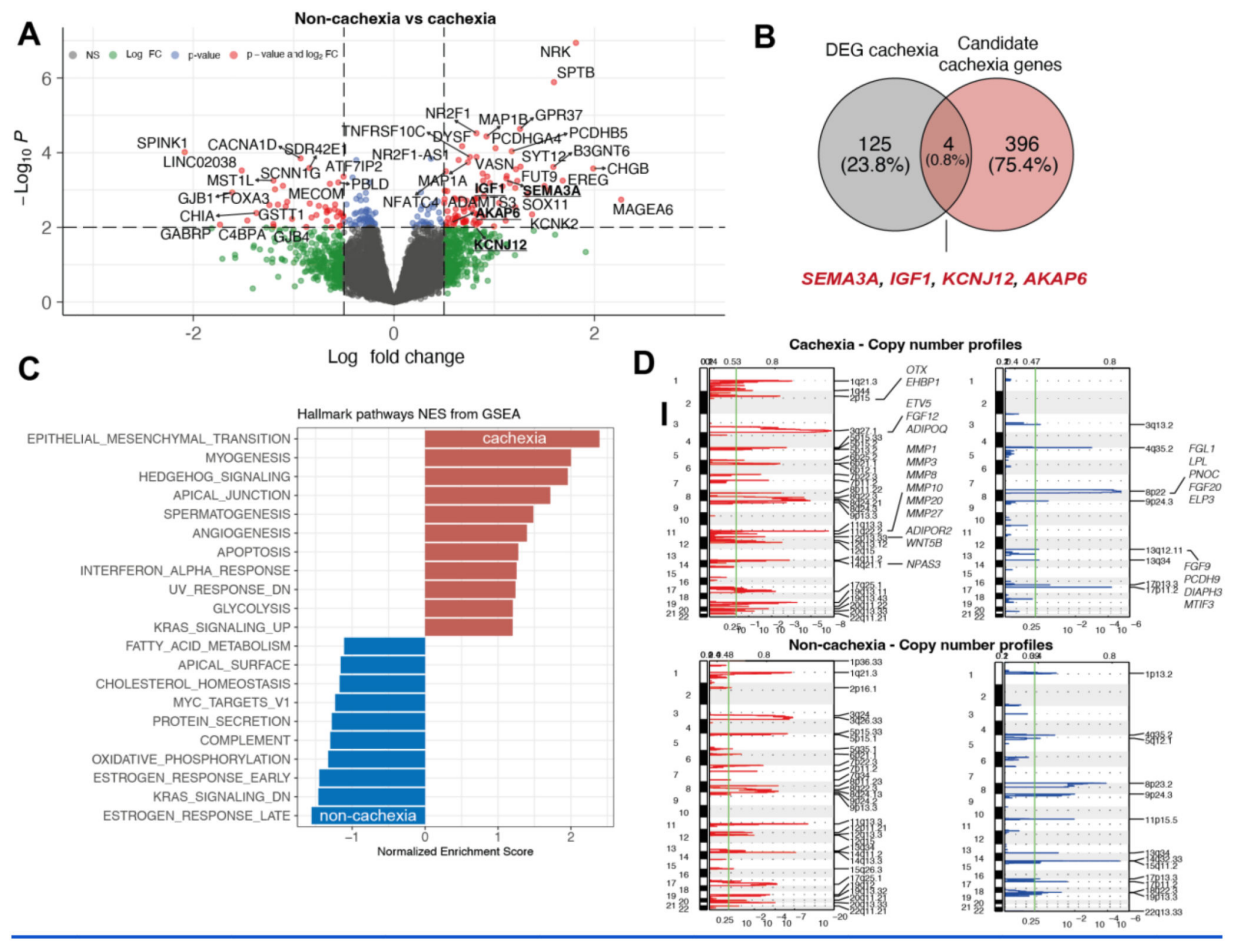
Tumour genomic and transcriptomic profiles according to cancer cachexia and non-cachexia groups. **A** Tumour differential gene expression between patients in the cachexia versus non-cachexia groups, adjusted for number of tumour regions, sex, and histology. **B** Overlap of differentially expressed genes between the cachexia group and candidate cachexia genes. **C** Hallmark gene set enrichment in the cachexia (red) versus non-cachexia groups (blue), adjusted for sex and histology. **D** GISTIC analysis of copy number alterations of cachexia (upper row) and non-cachexia groups (lower row). Y-axes indicate chromosomal positions (1-22), red plots indicate gains, blue plots indicate losses. X-axes indicate q-values. Most significant peaks are indicated on the right of each panel; regions with FDR q≤0.25 (vertical green line) are considered significant. GISTIC G-Scores are plotted on top of each panel.

Distinct differential gene expression profiles, adjusted for histology and sex (see methods), were observed in primary tumours from patients in the CAC versus non-CAC group. Gene expression of Melanoma-Associated Antigen 6 *MAGEA6*, matrix metalloproteinases, such as*ADAMTS3* and transcriptional and cytoskeletal regulators, such as *NR2F1* and *SPTB*, were significantly increased in the CAC group (Figure 3 A). To explore the biological relevance of these differentially expressed genes in the development of CAC, we cross-referenced genes significantly associated with CAC with a list of 400 genes generated by reviewing supporting literature (hereafter referred to as ‘cachexia candidate gene list’). The genes from this list are known to be associated with the development of either obesity or cachexia in pre-clinical and clinical models, or derived from genome wide association studies of obesity, BMI, and cachexia (Table S9)^24 25–38^. From this gene list, Semaphorin-3A (*SEMA3A*), Insulin-like growth factor 1 (*IGF1*), potassium channel *KCNJ12*, and Akinase Anchoring Protein 6 (*AKAP6*) were found to be differentially upregulated in the CAC group (Figure 3 B).

Hallmark genesets significantly enriched in primary tumours from the CAC group included epithelial-mesenchymal transition, hedgehog signalling and myogenesis, interferon-alpha response and glycolysis, while oestrogen-response pathways, oxidative phosphorylation and fatty acid metabolism were more enriched in the non-CAC group (Figure 3 C). Differential gene expression profiles were also observed when considering losses for individual body composition compartments and body weight, suggesting possibly distinct wasting mechanisms specific to adipose and muscle tissue (Figure S6, S7).

Somatic copy number analysis was performed using GISTIC2.0 to identify amplifications and deletions specific to the CAC and non-CAC groups, and the SAT/VAT/SKM and body weight loss prognostic thresholds(Figure S8, Figure 3 D)^39^. Multiple loci from the cachexia candidate gene list were found to be exclusively amplified in the CAC group, including chromosome 11q22.3 containing various metalloproteinases, such as MMP1 and MMP3, and chromosome 3q27.1, containing ADIPOQ.

Since some inflammatory signalling pathways (interferon-alpha response) were observed to be upregulated in the CAC group, we further investigated whether this was concordant with increased inflammatory cell infiltration using the previously published TCRA algorithm, providing T-cell infiltration estimates based on whole-exome sequencing data ^40^. No significant difference in TCRA scores, i.e., T-cell infiltrates, was observed between primary tumours in the CAC and non-CAC group, and this remained so after adjusting for sex (Figure S9 A-C). However, TCRA scores in the blood were higher in the non-CAC group, suggesting higher circulating T-cell levels in the circulation (Figure S9 D-F). In addition, we used the Danaher immune signature ^41^ and CIBERSORTx^42^ RNAseq cell type deconvolution approaches to compare the abundance of inflammatory cells in tumours in the CAC and non-CAC groups, but no difference was observed (Figure S10 and Figure S11).

In summary, the analysis of tumour whole exome and RNA sequencing data in patients who did and did not develop CAC revealed distinct genomic and transcriptomic profiles suggesting defined mechanisms and mediators of cachexia, including upregulated inflammatory signalling or increased expression and amplification of matrix metalloproteinases.

### Associations between circulating proteins and cancer cachexia features

**Figure 4:**
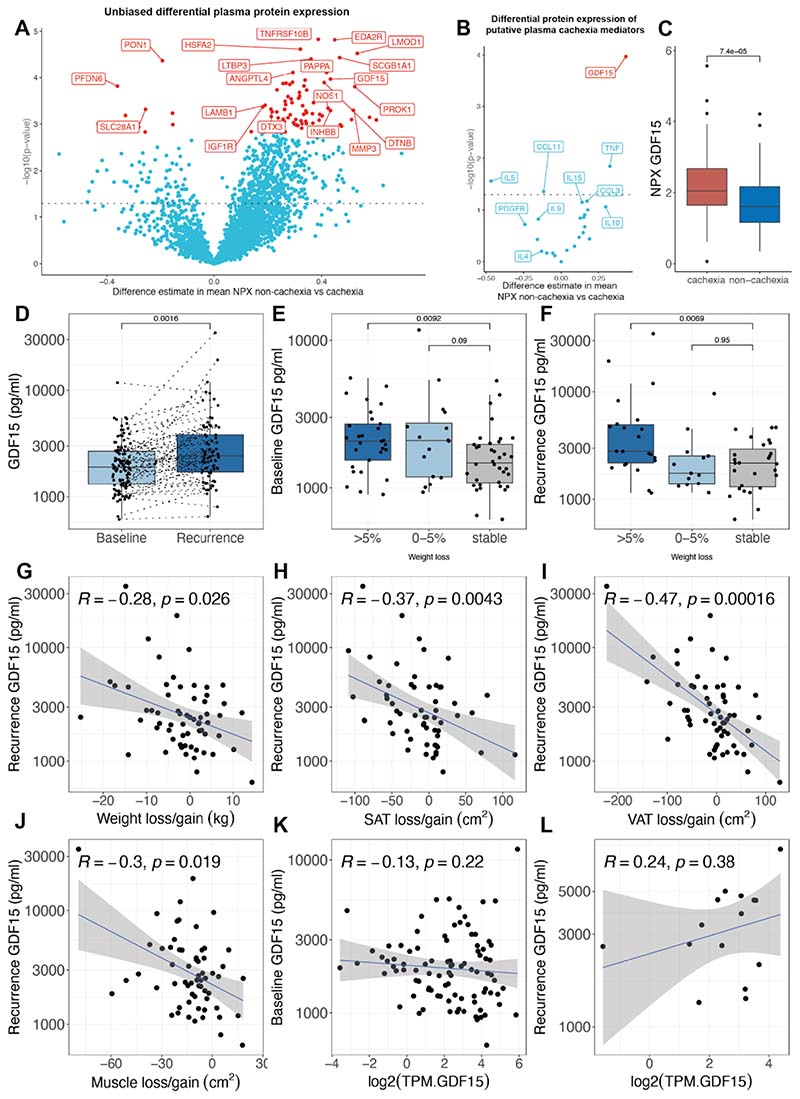
Differential protein expression and associations between circulating GDF15, body composition and body weight changes, and cancer cachexia. **A** Differential plasma proteome of patients in the non-cachexia versus cachexia groups. Red labels indicate significant differential protein expression after adjusting for multiplicity. **B** Differential plasma protein expression of putative cachexia mediators in the non-cachexia versus cachexia group. **C** Normalized plasma protein expression of GDF15 in the non-cachexia versus cachexia groups (two-sided Wilcoxon test). **D** Plasma GDF15 levels in patients at diagnosis (baseline) or first recurrence of NSCLC in the TRACERx cohort (two-sided Wilcoxon test). **E** Baseline and **F** recurrence GDF15 levels according to weight change category in the TRACERx cohort (two-sided Wilcoxon test, error bars indicate standard deviation). **G-J** Spearman correlation of recurrence GDF15 levels and loss/gain of body weight (N=62)(**G**), subcutaneous adipose tissue (SAT)(**H**), visceral adipose tissue (VAT) (**I**), and muscle (**J**)(N=61). **KL** Spearman correlation of baseline (**K**) and recurrence (**L**) GDF15 levels and GDF15 gene expression as transcripts per million (TPM). All Wilcoxon tests are two-sided and box plots represent lower quartile, median and upper quartile, whiskers extend to a maximum of 1.5 × IQR beyond the box. Points indicate individual data points. Grey shade areas represent 95% confidence intervals. Y-axes represent log10 scales.

Given the systemic implications of CAC and the possible role of circulating mediators in driving this phenotype, plasma samples collected at diagnosis (141 patients: 55 in CAC, 70 non-CAC group) and at relapse (115 patients, 43 in CAC, 58 in non-CAC group) were subjected to unbiased proteomic profiling using the Olink Explore 3072 platform to investigate the presence of significant proteins associated with CAC ^43^(Figure S2). Differential plasma protein expression was observed to varying extents between the different groups of SAT, VAT, SKM and BMI-adjusted weight loss (Figure S12). 79 proteins, including TRAIL2 receptor 2 (TNFRSF10B), EDA2R and HSPA2, demonstrated increased expressed in the CAC group (Figure 4 A), whereas 9 proteins, including SLC28A1, MENT and PDE4D, were more expressed in the non-CAC group (Table S10). Concordant increased tumour gene expression and plasma protein expression was only found for *HSPA2* and *KIAA0319* in the CAC group (Table S10).

Of 24 circulating proteins previously reported to play a role in CAC^44^ (Table S11) only CCL11, IL5, TNF and GDF15 showed higher expression in plasma from patients with CAC. Of these, only GDF15, a known mediator of anorexia and weight loss^45^, was significantly differentially increased (Benjamin-Hochberg correction) (Figure 4B). The normalised plasma protein expression of GDF15 was significantly higher in the CAC group compared with the non-CAC group (p<0.001) (Figure 4 C). Furthermore, a significant correlation between normalised protein expression of GDF15 and loss of SAT, VAT, SKM and body weight at relapse, was observed (Figure S13).

This relationship between increased GDF15 and the CAC phenotype was also seen using an alternative method with an ELISA-based GDF15 assay (Roche Elecsys GDF15) in a cohort of TRACERx patients for whom plasma at diagnosis (107 patients) and at relapse (89 patients) was available (Figure 4 D). The median circulating GDF15 levels in this cohort were 1902 pg/ml (normal range 200-1200 pg/ml^45^) at diagnosis, and further increased at relapse (median 2393.5 pg/ml) (Figure 4 D). Notably, serum GDF15 levels in the TRACERx cohort (median age 70 years) were higher than previously published in age-matched non-cancer volunteers (60-70 years, median plasma GDF15 levels of 866 pg/ml) and in keeping with previous reports of increased circulating GDF15 in patients with NSCLC ^46–49^. Median GDF15 levels of patients in the CAC group were overall significantly higher (2483 pg/ml) compared to 1756 pg/ml in the non-CAC group (p<0.001) (Figure S14). Circulating GDF15 levels were significantly associated with increased age at diagnosis and the squamous cell carcinoma histology subtype. There was no significant association between GDF15 levels and BMI at diagnosis, smoking status or number of pack years, use of adjuvant treatment, tumour stage or volume (Figure S15). GDF15 at diagnosis and relapse levels were not associated with time to recurrence in a Cox regression analysis (HR 1.0, 95% CI 1.0-1.0, p=0.269 and p=0.350, respectively).

Circulating GDF15 levels, at diagnosis and at relapse, were significantly higher in patients who developed grade 4 BMI-adjusted weight loss at relapse compared to those patients who remained stable or gained weight (grade 0) (Figure 4 E, F). Based on body composition analysis, increased circulating GDF15 was significantly associated with loss of body weight, as well as SAT, VAT and SKM tissue (Figure 4 G-J, Figure S15). Whole exome and RNA sequencing data were analysed to investigate whether increased circulating GDF15 was associated with genomic alterations and/or increased gene expression in the primary tumour. Mutations in the GDF15 gene were found in only one patient (c.A313G: p.I105V). There was no significant correlation between GDF15 gene expression and circulating GDF15 levels (Figure 4 K, L), although the power to detect any correlation was limited by the small number of relapse samples in this cohort. Furthermore, the ploidy-adjusted copy number of the GDF15 gene on chromosome 19p13.11q did not correlate with gene expression (Figure S17A) or with circulating GDF15 levels (Figure S17B). However, GDF15 copy number gains in relapsed tumour tissue, observed in 8 patients, were associated with higher circulating GDF15 levels compared to tumours with copy number losses (p=0.045, Figure S17C).

To explore whether the relationship between increased circulating GDF15 and the development of CAC was restricted to early-stage NSCLC, we measured plasma GDF15 levels in 164 patients with stage IV lung adenocarcinoma in an independent cohort of patients with metastatic NSCLC (NCT01360554)^50^. Circulating GDF15 levels were significantly higher in patients with grade 3 and 4 BMI-adjusted weight loss compared to patients with grade 0 BMI-adjusted weight loss, i.e. stable body weight (Figure S18), suggesting a similar relationship between circulating GDF15 and weight loss in the advanced disease setting.

Overall, these data suggest that patients who develop features of CAC at first relapse have distinct tumour genomic and transcriptomic, as well as plasma proteomic, profiles. Among these, circulating GDF15 showed the strongest correlation with loss of adipose, as well as skeletal muscle, tissue and body weight, emphasising the potential of GDF15-targeted therapy in the management of CAC.

## Discussion

This study integrates longitudinal body composition with matched tumour whole exome and RNA sequencing data, as well as plasma proteomic data in a combined analysis of the biological correlates associated with the development of CAC. Our first aim was to generate body composition profiles of patients with early stage lung cancer and to elucidate their association with survival outcomes. At the time of cancer diagnosis, low SAT, VAT and SKM as measures of body composition were each associated with poor LCSS and OS in both the TRACERx and BLCS cohorts. ^51,52^. Whilst low SAT has previously been shown to be associated with shorter OS in patients with cancer ^53^, to the best of our knowledge we demonstrate here for the first time that low VAT is independently associated with both OS and LCSS at the time of diagnosis in early stage NSCLC.

Two distinct features of CAC are the loss of body weight and skeletal muscle ^6^. The second aim of this study was to examine how body composition and body weight change in the context of lung cancer recurrence. We observed that most patients experienced a loss across all three body compartments, but small subgroups of patients with pronounced or isolated loss of SAT, VAT or SKM were also identified, suggesting the possibility that distinct CAC clinical phenotypes may exist whereby individual, or a combination of, body composition compartments may be preferentially affected ^54^. Importantly, body composition measurements were made using an established deep learning-based algorithm to avoid investigator-dependent variation in the datasets between diagnosis and relapse ^21,55^.

We created a definition of CAC based on the observed associations between each individual measure and LCSS and OS. Our preliminary categorisation of CAC consisted of patients who had any of ≥20% loss in SAT, ≥20% loss in VAT, ≥10% loss in SKM, or grade 4 BMI-adjusted weight loss between diagnosis and relapse. Future analyses of all four measures would further explore their impact on LCSS and OS including potential interactions between them, to redefine CAC using prognostic models that can be validated in independent patient cohorts.

The third aim of this study was to explore potential molecular mechanisms and mediators of CAC. Using our study-defined categorisation of CAC, we analysed the genomic and transcriptomic profiles of tumours in patients with and without CAC, to investigate the presence of alterations associated with CAC, albeit restricted to the primary, as opposed to relapse, tumour. By cross-referencing the differential gene expression profiles generated from bulk tumour analysis with a list of 400 genes derived from published reports relating to obesity and cachexia, we identified Semaphorin-3A (*SEMA3A*), Insulin-like growth factor 1 (*IGF1*), the potassium channel *KCNJ12*, and A-kinase anchor protein 6 (*AKAP6*) to have increased differential expression in the CAC group ^56^. Additionally, we observed differential upregulation of multiple inflammatory pathways in the CAC group and detected higher expression levels of several genes of interest, including lipopolysaccharide binding protein (*LBP*) and metallopeptidases, such as *ADAMTS*3 compared to patients in the non-CAC group ^57,58^. This is in keeping with previous observations that have suggested tumour-driven systemic inflammation is a contributor to CAC ^59,60^. While several pre-clinical studies have reported increased tumour expression of a range of purported mediators of cachexia, such as IL17 in the murine Lewis Lung Carcinoma model and PTH-related protein^61,62^, we did not see significant differential expression of these genes in the primary tumours between the CAC and non-CAC groups.

The analysis of tumour copy number profiles in primary tumours revealed distinct copy number alterations in the CAC group. These included amplification of a cluster of MMPs on chromosome 11q22.3, including MMP1 and MMP3, which have been previously associated with tumour-induced muscle loss in drosophila models ^32^, as well as amplifications involving ADIPOQ (Adiponectin) on chromosome 3q27.1. High levels of Adiponectin, a regulator of insulin sensitivity and lipid metabolism, have previously been associated with low body weight and weight loss, while low levels have been associated with obesity ^63^. Overall, our transcriptomic and genomic analyses suggest specific tumour-derived factors may play a role in the development of CAC.

To study the presence of potential circulating mediators of the cachexia phenotype, we profiled the plasma proteomes of 115 patients both at diagnosis and relapse. Significant differential plasma protein expression between CAC and non-CAC groups was identified for several candidate mediators of cachexia. These included MMP3, with the corresponding gene also found to be amplified in the primary tumour, and the proinflammatory cytokine TNF-α. The most differentially, highly expressed, candidate plasma protein among patients with CAC was GDF15; a highly conserved member of the Transforming Growth Factor ß (TGF- ß) superfamily which circulates at physiologically low levels in healthy states. GDF15 expression and secretion is upregulated in response to cellular stress ^64,65^, and elevated circulating levels have been identified in a broad range of human diseases, including cardiac, renal, and respiratory failure, and notably anorexia and weight loss ^66–69^. There is mounting pre-clinical evidence that GDF15 may be a putative druggable target; with transgenic mice overexpressing GDF15 developing a cachexia-like syndrome that can be readily reversed with neutralising anti-GDF15 monoclonal antibodies ^67,70^.

We used an orthogonal GDF15-specific serological assay, demonstrating that circulating GDF15 levels correlated significantly with loss of SAT, VAT, SKM and body weight, suggesting a cachexia-mediating role for GDF15 in patients with NSCLC. The same association between circulating GDF15 levels and body weight loss was observed in an independent cohort of advanced metastatic NSCLC, suggesting that this association is agnostic of disease stage, and further corroborating the key role of GDF15 as a mediator of CAC in NSCLC. No clear evidence of increased tumour GDF15 expression was observed and no significant genomic amplification of the GDF15 locus was detected, however, GDF15 is also known to be produced in diverse tissue sites, including liver and kidney, which may act as alternative potential sources of pathological secretion. ^65,71^. Furthermore, GDF15 is subject to differential rates of production and clearance that are mediated by other factors, such as hepatic stabilin-1 and -2 ^72^.

While our study provides a NSCLC dataset integrating tumour genomics and plasma proteomics with body composition and body weight, there are limitations in the interpretation of the data. Firstly, the body composition analysis focuses on two timepoints in the disease course: diagnosis and first relapse. Conceivably, the cachexia phenotype may develop at subsequent time points of disease progression with increasing burden of disease and warrants further investigation. Moreover, our description of the CAC phenotype is based on changes that occur between diagnosis and first relapse, whereas the tumour genomic and transcriptomic data were mostly derived from the resected primary tumour. As such, our analyses are correlative and hypothesis-generating and therefore further studies to validate our findings, including functional experiments, are warranted. A selection bias cannot be excluded in the plasma proteomic analyses, since only patients with available complete baseline and relapse imaging data and sufficient amounts of banked plasma were analysed. Future studies aiming to establish the underlying biological mechanisms of CAC would benefit from incorporating functional patient data, such as physical activity, food intake and muscle function, as well as quality of life measures, to reflect the complexity of CAC and to capture its related constitutional symptoms. Finally, the identified thresholds used in this study to stratify patients into CAC and non-CAC groups are applicable to the TRACERx cohort alone, and their use in other studies would require further validation.

Overall, this study demonstrates the significant, independent, and potentially prognostic impact of altered body composition on clinical outcomes (LCSS and OS) in NSCLC. The presence of specific body composition changes, either predominant loss of adipose or muscle tissue, in subgroups of patients suggests distinct clinical subtypes of CAC, which may be driven by unique biological mechanisms warranting further investigation. We show that automated pipeline technologies unlock the potential to leverage CT imaging embedded in medical oncological practice to identify patients at risk of developing CAC, simultaneously providing the scientific means to study potential drivers and mechanisms of cachexia pathophysiology. Amidst the plethora of proposed pro-cachectic mediators, GDF15 emerges as a differentially expressed, and clinically measurable, protein, with a mounting evidence base establishing its potential to translate to a biotherapeutic target.

## Methods

### Patient cohorts

#### TRACERx cohort

TRACERx is a UK-wide prospective multi-centre study of patients with primary NSCLC that aims to define evolutionary trajectories for lung cancer through multiregion and longitudinal tumour sampling ^17^(NCT01888601). The study was approved by an independent Research Ethics Committee (13/LO/1546). All patients provided written informed consent. Patients are followed for up to 5 years from the point of primary diagnosis, through surgical resection to cure, cancer progression(s) and death. The study collects longitudinal clinical, epidemiological, and imaging data as well as multi-region tumour tissue samples. The study protocol with inclusion/exclusion criteria has been published previously.^17^

For the body composition and cachexia study, patients were included if a pre-operative abdominal CT incorporating the third lumbar vertebrae and performed within 3 months of primary surgical tumour resection, was available. For the delta cohort, all patients with available abdominal CT at the time of disease recurrence were included. Overall, 651 patients with a pre-surgery baseline CT were included, of which 188 had a relapse with corresponding CT scan.

#### BCLS cohort

Patients from the US cancer cohort in this analysis are part of the ongoing Boston Lung Cancer Study (BLCS), a multi-institutional epidemiology cohort study at MGB, and the DFCI. Inclusion criteria for this analysis were pathology confirmed diagnosis of lung cancer with available abdominopelvic CT or PET/CT scans within 4 months before and the 2 months after diagnosis, and relevant, a priori defined clinical covariates available. Patients were excluded if imaging or clinical data were incomplete or missing. The study was approved by the institutional review boards of all institutions and the requirement for written informed consent was waived. Information regarding smoking status was prospectively collected in the TRACERx study and was also available for all the patients in the BLCS cohort. Patient demographics are provided in Table 1.

#### ARCHER1009 cohort

ARCHER1009 was a randomised phase 3 study for patients with advanced NSCLC who were randomised to the EGFR inhibitors dacomitinib versus erlotinib.^50^ This study received ethical approval from the Pfizer Institutional Review Board and had been conducted in accordance with the Declaration of Helsinki (ClinicalTrials.gov Identifier: NCT01360554). For the cachexia study, 164 patients with available plasma and body weight data at two timepoints within 6 months were included. Classification of weight loss was similar to published schemes ^6,73^ and defined as either weight stable/gain, >0-5% weight loss, and >5% weight loss. Any prior treatment (chemotherapy, radiation, or surgery) must have been completed at least 2 weeks prior to randomization at the start of the study.

### Body composition and body weight measurements

For the TRACERx cohort, subcutaneous adipose tissue, visceral adipose tissue, and skeletal muscle tissue were quantified as areas (cm^2^) localised to the third lumbar vertebrae (L3) level. CT-scans were generated according to standard local protocols at the participating sites (Table S12). L3 selection, image segmentation and tissue area quantification were conducted via an automated deep learning-based pipeline (DAFS platform, Voronoi Health Analytics, Vancouver, British Columbia, Canada ^21,55,74,75^). To measure SAT, VAT and SKM areas at the L3 level, analyses were run with the ‘avg-L3mid[3]’ command, which measures across 3 slices above and below the midst of L3 in order to increase data accuracy (Figure S19). The following Hounsfield unit (HU) boundaries were used: For SAT -190 to -30 HU, for VAT -150 to -50 HU and for SKM -29 to 150 HU. Accurate L3 selection and segmentation quality was manually inspected for all patients by an experienced investigator (medical oncologist with over 10 years experience). To this end, each CT annotation and segmentation was inspected via a sagittal, coronal and axial view of each scan using the ‘quickcheck’ option; mis-annotations was corrected using the CAST (CT Annotation and Segmentation Tool) feature from DAFS. To confirm the highly reproducible nature of the algorithm, 60 CT-scans were re-run twice through the automated annotation, segmentation and measurements steps. Using the precision metrics previously proposed by Arribas et al, ≤0.01% variance was observed between the runs (Table S13) ^76^. Absolute (cm^2^) change between baseline and relapse was calculated for SAT, VAT and SKM per patient.

For the independent BLCS cohort, baseline body composition was measured by a previously developed automated system ^22,77^, which produced body composition measurements at the L3 level for SAT, VAT and SKM in cm^2^. Manual quality control was conducted for all images in the BLCS cohort by an experienced investigator (radiologist with over 10 years experience).

Body weight data in the TRACERx, BCLS and ARCHER1009 cohorts were collected from medical records in routine health care settings; scale calibrations and type of clothing was conducted according to local standards. For the TRACERx cohort, body weight changes were assessed between baseline and relapse; for the ARCHER1009 cohort, body weight changes were assessed between time of enrolment and end of study. BMI-adjusted weight change was calculated as follows, similar to previous publications ^23^:

### Whole exome and RNA sequencing

Whole exome sequencing was performed on DNA purified from tumour tissue with matched germline from whole blood, as described previously ^17,78^. RNA was extracted from primary tumour tissue, and downstream analyses were conducted as reported previously; where applicable, multi-region gene expression data were handled as average per tumour or adjusted by a linear-mixed effects model, also accounting for histology and sex (Martinez-Rui et al, 2023). P value <0.01 and absolute log folder change >1 was used to identify differentially expressed genes. Gene set enrichment analysis (GSEA) was done by pre-ranking the genes by fold-change and using the fgsea R package (v1.22.0) and using the hallmark gene set from the Molecular Signatures Database (v.7.4) with a minimal size of 15 and maximal size of 500. GISTIC2.0 was used to analyse copy number alterations in tumour tissues according to cachexia and non-cachexia group ^39^.

A list of candidate cachexia genes, i.e. genes possibly associated with cachexia, was generated by reviewing supporting literature, including genome wide association studies on obesity and cachexia, since both extremes can share common metabolic perturbations, as well as murine and drosophila models (see Table S9).

### Plasma proteomics

Plasma proteomes were profiled using the Olink Explore 3072 platform (Olink, Uppsala, Sweden) at Bioxpedia (Aarhus, Denmark) following the standard Olink-certified protocol.^43^ TRACERx plasma samples and control samples were plated on three 96 well plates that were processed in one batch. For data analysis, protein expression as log2 of normalized protein expression (NPX) was used. Comparisons of protein expression between body composition groups were made by Welch 2-sample t-tests with Benjamin-Hochberg correction. Data that did not pass the Olink-specified quality control metrics were excluded from the analysis. R packages OlinkAnalyze v3.1.0 and ggplot2 v3.3.6 were used for data analysis and visualization.

For the orthogonal GDF15 validation, GDF15 was measured in TRACERx plasma samples using the Elecsys GDF15 immunoassay (F. Hoffmann-La Roche, Basel, Switzerland) at baseline ahead of surgery and at diagnosis of relapse. Plasma samples from the ARCHER1009 samples were obtained at the end of study and human GDF15 was measured using ELISA (R&D Systems, Minneapolis, MN).

### Statistical analyses

All statistical analyses were performed in R v4.0.3. GDF15 plasma levels were log10 transformed. Tests involving correlations were done using stat_cor from the R package ggpubr (v04.0) with linear regression and Spearman’s rank-order correlation. Categorical comparisons were made by ANOVA, student’s t-test, or Wilcoxon test. P-values were two-sided and considered as statistically significant if below 0.05. Plotting and analysis in R was also done by ggplot2 (v3.3.3), cowplot (v1.1.1), tidyr (v1.1.1) and dplyr (v1.0.4). For LCSS, an event was death from lung cancer; and all other patients were censored at the date last seen alive or death from other causes (competing risk analyses, in which death from other causes were treated as a competing event, did not produce materially different associations). For OS, an event was death from any cause; and all other patients were censored at the date last seen alive.

## Supplementary Material

Supplementary Material

## Figures and Tables

**Table:** 

	Body mass index (kg/m^2^)				
Weight loss (%)	≥28	25-27.9	22-24.9	20-21.9	<20
±2.4	0	0	1	1	3
2.5-5.9	1	2	2	2	3
6-10.9	2	3	3	3	4
11-14.9	3	3	3	4	4
≥15	3	4	4	4	4

**Table 1:** Baseline patient characteristics of TRACERx and BLCS cohorts. SD standard deviation, BMI body mass index, VAT visceral adipose tissue, SAT subcutaneous adipose tissue, SKM skeletal muscle

	TRACERx (N=651)	BLCS (N=420)
**Age**, Mean (SD), years	68.7 (9.12)	64.53 (10.1)
**BMI**, mean (SD), kg/m^2^	26.7 (5.11)	26.48 (5.4)
**Weight,** mean (SD), m	75.0 (16.9)	75.02 (18.2)
**Height**, mean (SD), m	1.67 (0.1)	1.68 (0.1)
**VAT baseline**, mean (SD), cm^2^	138 (98.7)	145.5 (113.9)
**SAT baseline**, mean (SD), cm^2^	169 (94.3)	190.5 (103.5)
**SKM baseline**, mean (SD), cm^2^	126 (34.5)	133.6 (33.9)
**Sex**		
Female	286 (43.9%)	198 (47.1)
Male	365 (56.1%)	222 (52.9)
**Ethnicity**		
White-Caucasian		411 (97.9)
African American		9 (2.1)
Non-White	36 (5.5%)	
White-British-Irish	590 (90.6%)	
White-Other	22 (3.4%)	
Missing	3 (0.5%)	
**Smoking status**		
Current Smoker	89 (13.7%)	155 (36.9)
Ex-Smoker	513 (78.8%)	216 (51.4)
Never Smoked	49 (7.5%)	49 (11.7)
**NSCLC stage**		
IA	131 (20.1%)	203 (48.3) - all stage I
IB	139 (21.4%)	
IIA	124 (19.0%)	63 (15.0) - all stage II
IIB	113 (17.4%)	
IIIA	138 (21.2%)	154 (36.7) - all stage III
IIIB	6 (0.9%)	
**Histology**		
Adenocarcinoma	362 (55.6%)	223 (53.1)
Squamous cell carcinoma	211 (32.4%)	104 (24.8)
Other	65 (10.0%)	93 (22.1)
N/A	13 (2.0%)	
**Adjuvant treatment**		
Adjuvant	227 (34.9%)	229 (54.5)
No adjuvant	408 (62.7%)	191 (45.5)
Missing	16 (2.5%)	

## Data Availability

The RNA sequencing and Whole Exome Sequencing data (from the TRACERx study) generated, used or analysed during this study are available through the Cancer Research UK & University College London Cancer Trials Centre (ctc.tracerx@ucl.ac.uk) for academic non-commercial research purposes only, subject to review of a project proposal that will be evaluated by a TRACERx data access committee and any applicable ethical approvals, and entering into an appropriate data access agreement. All code to reproduce figures will be made available at publication or upon request from reviewers.
