# Supplementary material for: Body composition and lung cancer-associated cachexia in TRACERx
Source: Nat Med. Author manuscript; Available in PMC 2023 Apr 25. (PMC7614477; doi:10.1038/s41591-023-02232-8)
Supplement: Supplementary Material [file EMS174138-supplement-Supplementary_Material.docx]

**SUPPLEMENTARY MATERIAL**

**Table of Contents**

[Table S1. Uni- and multivariable analysis of lung cancer-specific survival in TRACERx.](#_heading=h.tupot6dwky2d) 2

[Table S2. Uni- and multivariable analysis of overall survival in TRACERx.](#_heading=h.b0ibf8xv5luh) 2

[Table S3. Uni- and multivariable analysis of lung cancer-specific survival in BLCS.](#_heading=h.iyz7l1x6vow7) 3

[Table S4. Uni- and multivariable analysis of overall survival in BLCS.](#_heading=h.al437drm0a3x) 4

[Table S5. Sensitivity analysis with alternative body composition percentiles in TRACERx.](#_heading=h.piblqsg6vqn9) 4

[Table S6. LCSS according to body composition change subgroup in TRACERx.](#_heading=h.4a0ulqgeg93d) 5

[Table S7. Patient characteristics according to cachexia status.](#_heading=h.uqx8i0n1s16k) 6

[Table S8. Association between cachexia status and LCSS and OS.](#_heading=h.iemcv8y30bqs) 7

[Table S9. Candidate cachexia genelist.](#_heading=h.rruz4549807x) 7

[Table S10. Differentially expressed plasma proteins in patients with cachexia versus non-cachexia.](#_heading=h.iqv96uyhd8m1) 9

[Table S11. Circulating cachexia mediator captured by Olink assay.](#_heading=h.yau17v6ayuoc) 11

[Table S12. Technical information on local CT imaging analysis.](#_heading=h.6ddbx8tglxnv) 12

[Table S13. Body composition precision metrics.](#_heading=h.l3ynuzye2lag) 1

[Figure S1. Correlation between body composition metrics and body weight.](#_heading=h.nlz73fcg73jc) 2

[Figure S2. Patient and sample selection in TRACERx.](#_heading=h.o2svdrgor28l) 3

[Figure S3. Identification of prognostically relevant threshold of SAT, VAT and SKM loss.](#_heading=h.4y88k2izkgjy) 3

[Figure S4. Survival according to SAT, VAT, SKM and BMI-adjusted weight loss.](#_heading=h.8524t3ifh23d) 4

[Figure S5. Lung cancer-specific survival and disease-free survival according to cachexia status.](#_heading=h.v34aux3mqzob) 5

[Figure S6. Differential gene expression according to SAT, VAT, SKM and body weight loss.](#_heading=h.k2jw223osps5) 6

[Figure S7. Gene set enrichment according to SAT, VAT, SKM and body weight loss.](#_heading=h.ndk19jlxje3z) 7

[Figure S8. GISTIC copy number profiles according to SAT, VAT, SKM and body weight loss.](#_heading=h.ds536jlioef) 8

[Figure S9. TCRA scores according to CAC and non-CAC groups.](#_heading=h.l2e5m6buxvdq) 9

[Figure S10. Danaher scores according to CAC and non-CAC groups.](#_heading=h.8olt5umj698i) 10

[Figure S11. Cell type deconvolution by CIBERSORTx according to CAC and non-CAC groups.](#_heading=h.mrybza4zpmr8) 11

[Figure S12. Differential plasma protein expression.](#_heading=h.v0s2ubiusfdb) 12

[Figure S13. Correlation between plasma GDF15 expression (NPX) and changes in body composition and body weight.](#_heading=h.x8gyp5x8xai9) 13

[Figure S14. Plasma GDF15 levels in patients with versus without cachexia.](#_heading=h.hfloirbqmxph) 13

[Figure S15. Correlation of GDF15 plasma levels with clinical features.](#_heading=h.1fob9te) 14

[Figure S16. Baseline plasma GDF15 levels and body composition/weight changes.](#_heading=h.z2huq3wnf4cf) 15

[Figure S17. Copy number alterations of GDF15.](#_heading=h.gjdgxs) 16

[Figure S18. Plasma GDF15 levels and BMI-adjusted weightloss in the ARCHER1009 cohort.](#_heading=h.30j0zll) 16

[Figure S19. Correlation between mid L3 and average L3 body composition measurements.](#_heading=h.dun53vnxi8yx) 16

# Table S1. Uni- and multivariable analysis of lung cancer-specific survival in TRACERx.

SAT = subcutaneous adipose tissue, VAT = visceral adipose tissue, SKM = skeletal muscle, pct = percentile. * Adjusted for age, sex, BMI, smoking, stage, histological subtype, ethnicity, adjuvant therapy

| LCSS |  | No pts (events) | Univariable HR (95% CI) | Multivariable HR* (95% CI) |
| --- | --- | --- | --- | --- |
| SAT pct | 20%-80% | 391 (73) | Reference | |
|  | <20% | 130 (29) | 2.24 (1.55-3.22, p<0.001) | 2.09 (1.33-3.30, p=0.001) |
|  | >80% | 130 (48) | 1.29 (0.84-1.99, p=0.242) | 1.60 (0.95-2.69, p=0.079) |
|  | | | | |
| VAT pct | 20%-80% | 391 (73) | Reference | |
|  | <20% | 130 (46) | 2.15 (1.49-3.11, p<0.001) | 1.73 (1.10-2.72, p=0.019) |
|  | >80% | 130 (31) | 1.38 (0.91-2.10, p=0.132) | 1.54 (0.95-2.51, p=0.082) |
|  | | | | |
| SKM pct | 20%-80% | 393 (80) | Reference | |
|  | <20% | 128 (45) | 1.93 (1.34-2.78, p<0.001) | 1.44 (0.95-2.19, p=0.088) |
|  | >80% | 130 (25) | 0.93 (0.59-1.45, p=0.743) | 1.24 (0.76-2.02, p=0.379) |

# Table S2. Uni- and multivariable analysis of overall survival in TRACERx.

SAT = subcutaneous adipose tissue, VAT = visceral adipose tissue, SKM = skeletal muscle, pct = percentile. * Adjusted for age, sex, BMI, smoking, stage, histological subtype, ethnicity, adjuvant therapy

| OS |  | No pts (events) | Univariable HR (95% CI) | Multivariable HR* (95% CI) |
| --- | --- | --- | --- | --- |
| SAT pct | 20%-80% | 391 (132) | Reference | |
|  | <20% | 130 (58) | 1.48 (1.09-2.02, p=0.012) | 1.49 (1.02-2.16, p=0.037) |
|  | >80% | 130 (49) | 1.21 (0.87-1.68, p=0.257) | 1.26 (0.85-1.87, p=0.246) |
|  | | | | |
| VAT pct | 20%-80% | 391 (133) | Reference | |
|  | <20% | 130 (56) | 1.42 (1.04-1.94, p=0.028) | 1.38 (0.95-2.01, p=0.093) |
|  | >80% | 130 (50) | 1.23 (0.89-1.71, p=0.207) | 1.17 (0.81-1.71, p=0.398) |
|  | | | | |
| SKM pct | 20%-80% | 393 (135) | Reference | |
|  | <20% | 128 (63) | 1.60 (1.19-2.16, p=0.002) | 1.28 (0.91-1.78, p=0.151) |
|  | >80% | 130 (41) | 0.90 (0.63-1.28, p=0.557) | 1.16 (0.80-1.69, p=0.430) |

# Table S3. Uni- and multivariable analysis of lung cancer-specific survival in BLCS.

SAT = subcutaneous adipose tissue, VAT = visceral adipose tissue, SKM = skeletal muscle, pct = percentile * Adjusted for age, sex, BMI, smoking, stage, histological subtype, ethnicity, adjuvant therapy

| LCSS |  | No pts (events) | Univariable HR (95% CI) | Multivariable HR* (95% CI) |
| --- | --- | --- | --- | --- |
| SAT pct | 20%-80% | 250 (81) | Reference | |
|  | <20% | 85 (41) | 1.74 (1.20-2.54, p=0.004) | 1.97 (1.24-3.14, p=0.004) |
|  | >80% | 85 (27) | 0.90 (0.58-1.39, p=0.63) | 0.94 (0.56-1.60, p=0.83) |
|  | | | | |
| VAT pct | 20%-80% | 252 (90) | Reference | |
|  | <20% | 84 (32) | 1.18 (0.79-1.77, p=0.41) | 1.57 (0.98-2.53, p=0.06) |
|  | >80% | 84 (27) | 0.85 (0.55-1.31, p=0.46) | 0.83 (0.50-1.37, p= 0.47) |
|  | | | | |
| SKM pct | 20%-80% | 252 (95) | Reference | |
|  | <20% | 85 (33) | 1.18 (0.79-1.76, p=0.41) | 1.35 (0.86-2.12, p=0.19) |
|  | >80% | 83 (21) | 0.58 (0.36-0.83, p=0.03) | 0.53 (0.32-0.87, p=0.01) |

# Table S4. Uni- and multivariable analysis of overall survival in BLCS.

* Adjusted for age, sex, BMI, smoking, stage, histological subtype, ethnicity, adjuvant therapy

| OS |  | No pts (events) | Univariable HR (95% CI) | Multivariable HR* (95% CI) |
| --- | --- | --- | --- | --- |
| SAT pct | 20%-80% | 250 (131) | Reference | |
|  | <20% | 85 (64) | 1.71 (1.27-2.31, p<0.001) | 1.71 (1.18-2.49, p=0.005) |
|  | >80% | 85 (45) | 0.92 (0.65-1.29, p=0.62) | 0.99 (0.65-1.51, p=0.98) |
|  | | | | |
| VAT pct | 20%-80% | 252 (140) | Reference | |
|  | <20% | 84 (55) | 1.33 (0.97-1.82, p=0.08) | 1.75 (1.20-2.54, p=0.003) |
|  | >80% | 84 (45) | 0.92 (0.66-1.29, p=0.64) | 0.86 (0.58-1.28, p=0.47) |
|  | | | | |
| SKM pct | 20%-80% | 252 (147) | Reference | |
|  | <20% | 85 (58) | 1.36 (1.00-1.85, p=0.05) | 1.41 (1.00-2.01, p=0.05) |
|  | >80% | 83 (35) | 0.62 (0.43-0.90, p=0.01) | 0.63 (0.42-0.93, p=0.02) |

# Table S5. Sensitivity analysis with alternative body composition percentiles in TRACERx.

Patients were categorised into more extreme percentiles, i.e. lower 10% (<10%), middle 10-90%, and highest 10% (>90%)

* Adjusted for age, sex, BMI, smoking, stage, histological subtype, ethnicity, adjuvant therapy

| **LCSS** | | | | |
| --- | --- | --- | --- | --- |
|  |  | **No pts (events)** | **Univariable HR (95% CI)** | **Multivariable HR* (95% CI)** |
| **SAT pct** | 10%-90% | 523 (108) | Reference | |
|  | <10% | 64 (28) | 2.70 (1.78-4.09, p<0.001) | 2.36 (1.42-3.93, p=0.001) |
|  | >90% | 64 (14) | 1.18 (0.67-2.05, p=0.569) | 1.79 (0.92-3.47, p=0.085) |
| **VAT pct** | 10%-90% | 523 (112) | Reference | |
|  | <10% | 64 (27) | 2.13 (1.40-3.25, p<0.001) | 2.42 (1.45-4.06, p=0.001) |
|  | >90% | 64 (11) | 0.86 (0.46-1.61, p=0.643) | 0.82 (0.42-1.62, p=0.567) |
| **SKM pct** | 10%-90% | 523 (110) | Reference | |
|  | <10% | 64 (26) | 2.25 (1.47-3.46, p<0.001) | 1.70 (1.05-2.75, p=0.031) |
|  | >90% | 64 (14) | 0.99 (0.57-1.72, p=0.964) | 1.71 (0.93-3.14, p=0.085) |
| **OS** | | | | |
| **OS** |  | **No pts (events)** | **Univariable HR (95% CI)** | **Multivariable HR* (95% CI)** |
| **SAT pct** | 10%-90% | 523 (181) | Reference | |
|  | <10% | 64 (35) | 2.00 (1.39-2.87, p<0.001) | 2.04 (1.32-3.15, p=0.001)) |
|  | >90% | 64 (23) | 1.16 (0.75-1.79, p=0.509) | 1.33 (0.80-2.21, p=0.277) |
| **VAT pct** | 10%-90% | 523 (184) | Reference | |
|  | <10% | 64 (32) | 1.53 (1.05-2.23, p=0.025) | 1.88 (1.21-2.92, p=0.005) |
|  | >90% | 64 (23) | 1.11 (0.72-1.72, p=0.624) | 0.94 (0.58-1.51, p=0.784) |
| **SKM pct** | 10%-90% | 523 (183) | Reference | |
|  | <10% | 64 (37) | 1.92 (1.35-2.73, p<0.001) | 1.56 (1.06-2.30, p=0.023) |
|  | >90% | 64 (19) | 0.81 (0.50-1.29, p=0.372) | 1.23 (0.74-2.05, p=0.417) |

# Table S6. Lung cancer-specific survival according to body composition change subgroup in TRACERx.

* Adjusted for age, sex, BMI, smoking, stage, histological subtype, ethnicity, adjuvant therapy

| LCSS | No pts | Univariable HR (95% CI) | Multivariable HR* (95% CI) |
| --- | --- | --- | --- |
| No loss | 92 (42) | Reference | |
| VAT loss | 22 (12) | 1.94 (1.02-3.71,p=0.044) | 1.98 (0.98-4.00,p=0.056)) |
| SAT loss | 5 (3) | 0.93 (0.29-3.01,p=0.905) | 0.89 (0.25-3.14,p=0.855) |
| SAT + VAT loss | 14 (9) | 1.41 (0.68-2.89,p=0.353) | 1.58 (0.73-3.39,p=0.243) |
| SKM loss | 21 (13) | 1.69 (0.91-3.16,0.097) | 1.94 (0.99-3.83,p=0.055) |
| SKM + VAT loss | 8 (5) | 1.40 (0.55-3.55,p=0.475) | 1.30 (0.50-3.39,p=0.593) |
| SKM + SAT loss | 3 (3) | 3.39 (1.05-10.99,p=0.042) | 3.41 (0.94-12.36,p=0.062) |
| SKM + SAT + VAT loss | 23 (15) | 2.80 (1.54-5.07,p=0.001) | 2.60 (1.39-4.87,p=0.003) |

#

# Table S7. Patient characteristics according to cachexia status.

No statistically significant differences were observed between the cachexia and non-cachexia group for all variables (two sided Wilcoxon test).

|  | **cachexia (N=108)** | **non-cachexia (N=155)** |  |
| --- | --- | --- | --- |
| **Age,** mean, years (SD) | 71.0 (9.96) | 67.7 (8.67) |  |
| **BMI** |  |  |  |
| Mean (SD) | 26.4 (5.28) | 26.7 (5.18) |  |
| **Weight** |  |  |  |
| Mean (SD) | 73.9 (17.5) | 74.9 (17.1) |  |
| **Height** |  |  |  |
| Mean (SD) | 167 (10.2) | 167 (9.90) |  |
| **VAT baseline** |  |  |  |
| Mean (SD) | 145 (100) | 134 (100) |  |
| **SAT baseline** |  |  |  |
| Mean (SD) | 160 (91.8) | 169 (93.8) |  |
| **Muscle baseline** |  |  |  |
| Mean (SD) | 128 (39.3) | 125 (35.1) |  |
| **Sex** |  |  |  |
| Female | 41 (38.0%) | 71 (45.8%) |  |
| Male | 67 (62.0%) | 84 (54.2%) |  |
| **Ethnicity** |  |  |  |
| Non-White | 8 (7.4%) | 8 (5.2%) |  |
| White-British-Irish | 92 (85.2%) | 141 (91.0%) |  |
| White-Other | 8 (7.4%) | 6 (3.9%) |  |
| **Smoking status** |  |  |  |
| Current Smoker | 7 (6.5%) | 24 (15.5%) |  |
| Ex-Smoker | 94 (87.0%) | 120 (77.4%) |  |
| Never Smoked | 7 (6.5%) | 11 (7.1%) |  |
| **NSCLC stage** |  |  |  |
| IA | 14 (13.0%) | 24 (15.5%) |  |
| IB | 22 (20.4%) | 34 (21.9%) |  |
| IIA | 21 (19.4%) | 28 (18.1%) |  |
| IIB | 19 (17.6%) | 26 (16.8%) |  |
| IIIA | 31 (28.7%) | 40 (25.8%) |  |
| IIIB | 1 (0.9%) | 3 (1.9%) |  |
| **Histology** |  |  |  |
| Adenocarcinoma | 53 (49.1%) | 98 (63.2%) |  |
| Other | 18 (16.7%) | 17 (11.0%) |  |
| Squamous cell carcinoma | 37 (34.3%) | 40 (25.8%) |  |
| **Adjuvant treatment** |  |  |  |
| Adjuvant | 42 (38.9%) | 68 (43.9%) |  |
| No adjuvant | 66 (61.1%) | 85 (54.8%) |  |
| N/A | 0 (0%) | 2 (1.3%) |  |

# Table S8. Association between cachexia status and lung cancer-specific survival and overall survival.

Cachexia = ≥20% subcutaneous and/or visceral adipose tissue loss, and/or ≥10% skeletal muscle tissue, and/or grade 4 BMI-adjusted weight loss

Non-cachexia = <20% subcutaneous and/or visceral adipose tissue loss, and/or <10% skeletal muscle tissue, and/or grade 0-3 BMI-adjusted weight loss

* Adjusted for age, sex, smoking, stage, histological subtype, ethnicity, adjuvant therapy

| LCSS |  | No pts (events) | Univariable HR (95% CI) | Multivariable HR* (95% CI) |
| --- | --- | --- | --- | --- |
|  | | | | |
| Cachexia status | Non-cachexia | 164 (63) | Reference | - |
|  | Cachexia | 108 (72) | 2.45 (1.74-3.44, p<0.001) | 2.33 (1.64-3.32, p<0.001) |

| OS |  | No pts (events) | Univariable HR (95% CI) | Multivariable HR* (95% CI) |
| --- | --- | --- | --- | --- |
|  | | | | |
| Cachexia status | Non-cachexia | 164 (76) | Reference | |
|  | Cachexia | 108 (83) | 2.37 (1.73-3.24, p<0.001) | 2.26 (1.63-3.13, p<0.001) |

# Table S9. Candidate cachexia genelist.

Gene names referenced from ^1^ ^2-15^

| A2M | CCL5 | FCN1 | HIVEP1 | LHCGR | MMP3 | PRMT7 | SKAP1 | ZFP64 |
| --- | --- | --- | --- | --- | --- | --- | --- | --- |
| A2ML1 | CD109 | FCN2 | HS6ST3 | LIF | MMP8 | PTBP2 | SKP1P2 | ZNF142 |
| ACSL5 | CDC14C | FCN3 | hsa-miR-653 | LINC00615 | MON1A | PTPRQ | SLC39A8 | ZNF536 |
| ADAM23 | CDH7 | FGF1 | HSD17B12 | LINC00669 | MTCH2 | PZP | SLX4 | ZNF608 |
| ADCY9 | CHST8 | FGF10 | HSS00296402 | LINC00936 | MTIF3 | QPCTL | SMG6 | ZNF646 |
| ADIPOQ | CLIP1 | FGF11 | ICAM1 | LINC01239 | MYEOV | RAB27B | SPDEF | ZNF668 |
| ADIPOR2 | CLUAP1 | FGF12 | ICOS | LINC01288 | MYL2 | RABEP1 | SRR | ZNF704 |
| AGBL4 | CNTF | FGF13 | IFNG | LINC01485 | N29617 | RACGAP1 | STAG1 | ZSWIM6 |
| AKAP6 | COL4A1 | FGF14 | IFNGR1 | LINC01488 | NADK | RAI1 | STK36 |  |
| ALDH2 | COL4A2 | FGF16 | IGF1 | LMX1B | NAV1 | RALYL | STON1-GTF2A1L | |
| ALKAL2 | COL4A3 | FGF2 | IGLON5 | LOC100130673 | NCAM1 | RARB | STX1B |  |
| ALPG | COL4A4 | FGF20 | IGSF9 | LOC100287559 | NCAM2 | RASA2 | STXBP6 |  |
| ALPI | COL4A5 | FGF22 | IGSF9B | LOC101926897 | NEGR1 | RBBP6 | SWI5 |  |
| ALPK1 | COL4A6 | FGF3 | IHH | LOC101929563 | NEGR1 | RBFOX1 | TAL1 |  |
| ALPL | CPEB4 | FGF4 | IL10 | LOC284260 | NLRC3 | RBJ | TAOK2 |  |
| ALPP | CPNE4 | FGF5 | IL12 | LOC285762 | NPAS3 | RBM6 | TBX15 |  |
| ANKS6 | CRB1 | FGF6 | IL13 | LOC646736 | NPY | REEP3 | TCF7L2 |  |
| APOBR | CREB1 | FGF7 | IL15 | LPL | NR3C1 | RETN | TDRG1 |  |
| ARIH1 | CSF1 | FGF9 | IL17 | LRFN2 | NRXN2 | RIT2 | TFAP2B |  |
| ASB4 | CSF2 | FGL1 | IL18 | LRIT1 | NRXN3 | RPL27A | TGFA |  |
| B3GALNT2 | CSF3 | FHIT | IL1B | LRIT3 | NT5C2 | RQCD1 | TLR1 |  |
| BANK1 | CXCL10 | FIBCD1 | IL2 | LRP1B | NT5C2 | RSRC2 | TLR4 |  |
| BBS4 | CXCL12 | FIGN | IL4 | LRRN6C | NTM | SBK1 | TMEM160 |  |
| BCL2 | CXCL8 | FLJ35779 | IL5 | LSAMP | NTNG1 | SCARB2 | TMEM18 |  |
| BDNF | CYP17A1 | FOXO3 | IL6 | LTA | NUCKS1 | SCG3 | TMEM38B |  |
| BMP2 | CYP27A1 | FTO | IL7 | MAGI2 | NUDT3 | SDK1 | TNF |  |
| BMP5 | DCC | GAB2 | IL8 | MAP2K3 | NUP54 | SDK2 | TNFA |  |
| BMP6 | DHH | GABRA5 | IL9 | MAP2K5 | OLIG3 | SEC16B | TNFSF10 |  |
| BMP7 | DIAPH3 | GABRB3 | INO80E | MAPK3 | OPCML | SELP | TNNI3K |  |
| BMP8A | DMXL2 | GALNT10 | IRS1 | MC4R | OTX1 | SEMA3A | TTLL4 |  |
| BMP8B | DOC2A | GBE1 | ITIH4 | MC4R | PAGR1 | SEMA3B | UBE2E3 |  |
| BTRC | DSCAM | GCKR | KAT8 | MFAP4 | PAPLN | SEMA3C | USH2A |  |
| C3 | DSCAML1 | GDF15 | KCNJ11 | MIR548A2 | PARD3B | SEMA3D | USP37 |  |
| C4A | DUSP26 | GGNBP2 | KCNJ12 | MIR548X2 | PARK2 | SEMA3E | VASP |  |
| C4B | DYNAP | GNPDA2 | KCNK3 | MIR8081 | PAX2 | SEMA3F | VEGFA |  |
| C5 | EDA | GP2 | KCNQ1 | MMP1 | PAX6 | SEMA3G | VKORC1 |  |
| C9orf4 | EHBP1 | GPC1 | KCTD13 | MMP10 | PCDH9 | SEMA4A | VPS33A |  |
| C9orf93 | ELAVL4 | GPC2 | KCTD15 | MMP12 | PDE1C | SEMA4B | VWC2 |  |
| CADM1 | ELL | GPC4 | KIRREL1 | MMP13 | PDGFA | SEMA4C | WARS2 |  |
| CADM2 | ELP3 | GPC6 | KIRREL2 | MMP14 | PDGFB | SEMA4D | WBSCR16 |  |
| CALCR | EPB41L4B | GPR139 | KIRREL3 | MMP15 | PDK4 | SEMA4F | WNT4 |  |
| CASC20 | ERBB4 | GPRC5B | KLC1 | MMP16 | PDXDC1 | SEMA4G | WNT5A |  |
| CBLN1 | ETS2 | GPRC5B | KLF7 | MMP17 | PGPEP1 | SEMA6D | WNT5B |  |
| CCL11 | ETV5 | GRID1 | LAMA1 | MMP19 | PLCD4 | SETD1A | YPEL3 |  |
| CCL2 | FAIM2 | GRP | LAMA2 | MMP20 | PMS2L11 | SFXN2 | YPEL3 |  |
| CCL24 | FAM110C | HGF | LAMA3 | MMP24 | PMS2L3 | SGSM2 | ZAG |  |
| CCL26 | FAM57B | HHIP | LAMA4 | MMP25 | PMS2P5 | SH2B1 | ZBTB10 |  |
| CCL3 | FANCL | HIF1AN | LAMA5 | MMP26 | PNOC | SHH | ZCCHC8 |  |
| CCL4 | FBXL19 | HIP1 | LEP | MMP27 | PRKD1 | SIX5 | ZDBF2 |  |

# Table S10. Differentially expressed plasma proteins in patients with cachexia versus non-cachexia.

| **Name** | **UniProt** | **estimate** | **cachexia** | **noncachexia** | **statistic** | **p.value** | **conf.low** | **conf.high** | **Adj_pval** |
| --- | --- | --- | --- | --- | --- | --- | --- | --- | --- |
| TNFRSF10B | O14763 | 0.38725962 | 1.50401 | 1.11675038 | 4.46414003 | 1.48E-05 | 0.21597888 | 0.55854037 | 0.01794026 |
| EDA2R | Q9HAV5 | 0.44944797 | 1.90076 | 1.45131203 | 4.45349434 | 1.52E-05 | 0.25024512 | 0.64865082 | 0.01794026 |
| HSPA2 | P54652 | 0.3208602 | 0.905082 | 0.5842218 | 4.35444282 | 2.41E-05 | 0.17530663 | 0.46641376 | 0.01794026 |
| LMOD1 | P29536 | 0.53384844 | 2.095874 | 1.56202556 | 4.26486617 | 2.98E-05 | 0.28713925 | 0.78055762 | 0.01794026 |
| SCGB1A1 | P11684 | 0.46825815 | 0.853798 | 0.38553985 | 4.2237221 | 3.66E-05 | 0.24963412 | 0.68688218 | 0.01794026 |
| LTBP3 | Q9NS15 | 0.36053543 | 0.976064 | 0.61552857 | 4.20004467 | 3.91E-05 | 0.19133427 | 0.52973659 | 0.01794026 |
| PON1 | P27169 | -0.193896 | 0.277189 | 0.47108496 | -4.2022228 | 4.27E-05 | -0.2849828 | -0.1028091 | 0.01794026 |
| PAPPA | Q13219 | 0.41869671 | 0.903214 | 0.48451729 | 4.03905397 | 7.63E-05 | 0.21429203 | 0.62310139 | 0.02430005 |
| ANGPTL4 | Q9BY76 | 0.29329267 | 1.257725 | 0.96443233 | 4.03816494 | 7.69E-05 | 0.15006838 | 0.43651695 | 0.02430005 |
| GDF15 | Q99988 | 0.43261933 | 2.145187 | 1.71256767 | 3.95714789 | 0.00010735 | 0.21695921 | 0.64827946 | 0.02430005 |
| IFIT3 | O14879 | 0.33864374 | 0.373349 | 0.03470526 | 3.97367798 | 0.00011283 | 0.17015633 | 0.50713114 | 0.02430005 |
| CLSTN2 | Q9H4D0 | 0.29167542 | 0.860007 | 0.56833158 | 3.93371927 | 0.00012568 | 0.14521027 | 0.43814058 | 0.02430005 |
| DTNB | O60941 | 0.40956241 | 1.4464 | 1.03683759 | 3.89831576 | 0.00012765 | 0.20253892 | 0.61658589 | 0.02430005 |
| HRC | P23327 | 0.26781732 | 0.466188 | 0.19837068 | 3.90175231 | 0.00013152 | 0.13244255 | 0.4031921 | 0.02430005 |
| FGFBP3 | Q8TAT2 | 0.25694205 | 1.084621 | 0.82767895 | 3.89803763 | 0.00013376 | 0.12693347 | 0.38695063 | 0.02430005 |
| EDN1 | P05305 | 0.28140852 | 1.833901 | 1.55249248 | 3.90732177 | 0.00014017 | 0.13911597 | 0.42370107 | 0.02430005 |
| STC1 | P52823 | 0.334425 | 0.945125 | 0.6107 | 3.86116575 | 0.00015084 | 0.16366946 | 0.50518054 | 0.02430005 |
| PFDN6 | O15212 | -0.3611378 | -1.384734 | -1.0235962 | -3.8815652 | 0.00015242 | -0.5449064 | -0.1773691 | 0.02430005 |
| PROK1 | P58294 | 0.52492983 | -0.002404 | -0.5273338 | 3.84614785 | 0.00015699 | 0.25595673 | 0.79390294 | 0.02430005 |
| KIAA0319 | Q5VV43 | 0.2339779 | 0.566417 | 0.3324391 | 3.82525003 | 0.00018351 | 0.11322954 | 0.35472626 | 0.0259121 |
| CKAP4 | Q07065 | 0.34432193 | 1.025349 | 0.68102707 | 3.8215763 | 0.00018502 | 0.16647971 | 0.52216415 | 0.0259121 |
| YAP1 | P46937 | 0.31720562 | 1.052756 | 0.73555038 | 3.79112484 | 0.00020859 | 0.1520285 | 0.48238275 | 0.02788431 |
| ULBP2 | Q9BZM5 | 0.31798475 | 1.069984 | 0.75199925 | 3.74249433 | 0.00024924 | 0.15025615 | 0.48571335 | 0.0313285 |
| CCN5 | O76076 | 0.27503305 | 1.287615 | 1.01258195 | 3.7252398 | 0.00025566 | 0.12942308 | 0.42064301 | 0.0313285 |
| EPHA2 | P29317 | 0.31437007 | 0.895943 | 0.58157293 | 3.71314028 | 0.0002769 | 0.14724677 | 0.48149336 | 0.03249965 |
| EFNA1 | P20827 | 0.28655403 | 1.140942 | 0.85438797 | 3.70391255 | 0.00028731 | 0.13382629 | 0.43928177 | 0.03249965 |
| PTK7 | Q13308 | 0.26403966 | 0.876875 | 0.61283534 | 3.68723666 | 0.00032183 | 0.1224831 | 0.40559622 | 0.0349368 |
| SMOC1 | Q9H4F8 | 0.28971435 | 1.077973 | 0.78825865 | 3.64420676 | 0.00034419 | 0.13292053 | 0.44650818 | 0.0349368 |
| TXNDC5 | Q8NBS9 | 0.3697213 | 1.888501 | 1.5187797 | 3.64951044 | 0.0003445 | 0.16981261 | 0.56962999 | 0.0349368 |
| LTBR | P36941 | 0.26634764 | 0.825292 | 0.55894436 | 3.62708512 | 0.00037994 | 0.12137671 | 0.41131857 | 0.03691086 |
| LAMB1 | P07942 | 0.18970821 | 0.502324 | 0.31261579 | 3.61224854 | 0.00039153 | 0.08609286 | 0.29332356 | 0.03691086 |
| SIGLEC8 | Q9NYZ4 | 0.32940258 | 0.974871 | 0.64546842 | 3.60691986 | 0.00040161 | 0.14919192 | 0.50961324 | 0.03691086 |
| IGF1R | P08069 | 0.1809168 | 0.659198 | 0.4782812 | 3.59488526 | 0.00042529 | 0.08156982 | 0.28026378 | 0.03790268 |
| NOS1 | P29475 | 0.42387676 | 1.313473 | 0.88959624 | 3.56601772 | 0.00045443 | 0.18947197 | 0.65828155 | 0.03810245 |
| DTX3 | Q8N9I9 | 0.21151892 | 0.980652 | 0.76913308 | 3.56592915 | 0.00047667 | 0.09438691 | 0.32865092 | 0.03810245 |
| SLC28A1 | O00337 | -0.2563241 | 0.188315 | 0.4446391 | -3.5459776 | 0.00047951 | -0.3987996 | -0.1138486 | 0.03810245 |
| RSPO1 | Q2MKA7 | 0.26205359 | 1.778116 | 1.51606241 | 3.56171824 | 0.00048013 | 0.11679772 | 0.40730946 | 0.03810245 |
| MMP3 | P08254 | 0.51933877 | 0.991032 | 0.47169323 | 3.53330077 | 0.00050539 | 0.22957275 | 0.80910478 | 0.03810245 |
| INHBB | P09529 | 0.43285163 | 1.708099 | 1.27524737 | 3.53389848 | 0.00051085 | 0.19129127 | 0.67441199 | 0.03810245 |
| ACTA2 | P62736 | 0.34106424 | 1.530868 | 1.18980376 | 3.53266281 | 0.00051893 | 0.15059766 | 0.53153082 | 0.03810245 |
| HLA-E | P13747 | 0.21607649 | 0.573381 | 0.35730451 | 3.51970249 | 0.00054231 | 0.09497114 | 0.33718184 | 0.03810245 |
| TIMP1 | P01033 | 0.23309165 | 1.05053 | 0.81743835 | 3.51837629 | 0.00054414 | 0.10240567 | 0.36377764 | 0.03810245 |
| FUT3_FUT5 | P21217_Q1 | 0.3437324 | 1.027773 | 0.6840406 | 3.50714111 | 0.00056634 | 0.15039489 | 0.53706991 | 0.038307 |
| MENT | Q9BUN1 | -0.1561942 | -0.072301 | 0.08389323 | -3.4962755 | 0.00057959 | -0.2442805 | -0.068108 | 0.038307 |
| IL5RA | Q01344 | 0.36580463 | 0.321552 | -0.0442526 | 3.49551944 | 0.00059317 | 0.15933436 | 0.57227491 | 0.038307 |
| THBS2 | P35442 | 0.33313953 | 0.697626 | 0.36448647 | 3.49424481 | 0.00061383 | 0.14486517 | 0.5214139 | 0.038307 |
| PI3 | P19957 | 0.39273716 | 0.341074 | -0.0516632 | 3.49201989 | 0.00061467 | 0.17068356 | 0.61479076 | 0.038307 |
| EFEMP1 | Q12805 | 0.28316613 | 1.436615 | 1.15344887 | 3.47655486 | 0.00062521 | 0.12253667 | 0.44379558 | 0.038307 |
| PDE4D | Q08499 | -0.3310726 | -0.820026 | -0.4889534 | -3.4588414 | 0.00065134 | -0.519714 | -0.1424312 | 0.03885808 |
| FABP5 | Q01469 | 0.40204744 | 1.83687 | 1.43482256 | 3.45683125 | 0.00066063 | 0.17278113 | 0.63131376 | 0.03885808 |
| ADA | P00813 | 0.21285536 | 0.394411 | 0.18155564 | 3.45853778 | 0.0006809 | 0.09139599 | 0.33431474 | 0.0392655 |
| CALCA | P01258 | 0.57968007 | 1.330553 | 0.75087293 | 3.44047305 | 0.00071011 | 0.24738855 | 0.91197159 | 0.04000751 |
| PTX3 | P26022 | 0.27529556 | 0.514173 | 0.23887744 | 3.43323019 | 0.00072558 | 0.11717297 | 0.43341814 | 0.04000751 |
| CD274 | Q9NZQ7 | 0.23829706 | 0.694773 | 0.45647594 | 3.4379431 | 0.00073458 | 0.10148561 | 0.37510851 | 0.04000751 |
| AGRN | O00468 | 0.25648744 | 0.511313 | 0.25482556 | 3.42007974 | 0.00077977 | 0.10847263 | 0.40450224 | 0.04047262 |
| WFDC2 | Q14508 | 0.30355741 | 1.836392 | 1.53283459 | 3.40888458 | 0.00079004 | 0.12795314 | 0.47916168 | 0.04047262 |
| IL19 | Q9UHD0 | 0.51254105 | 0.67202 | 0.15947895 | 3.41863663 | 0.0007929 | 0.21652119 | 0.80856092 | 0.04047262 |
| MUC16 | Q8WXI7 | 0.6049124 | 1.707753 | 1.1028406 | 3.39811984 | 0.00081109 | 0.25399791 | 0.95582689 | 0.04047262 |
| PRG2 | P13727 | 0.39082595 | 2.037644 | 1.64681805 | 3.39878652 | 0.00081977 | 0.16405069 | 0.61760122 | 0.04047262 |
| ADAMTS16 | Q8TE57 | 0.32339576 | 1.113092 | 0.78969624 | 3.40243437 | 0.00084285 | 0.13569208 | 0.51109944 | 0.04047262 |
| LAIR1 | Q6GTX8 | 0.34080214 | 1.153948 | 0.81314586 | 3.39416266 | 0.00086908 | 0.14249675 | 0.53910752 | 0.04047262 |
| SCARA5 | Q6ZMJ2 | 0.2609566 | 0.586316 | 0.3253594 | 3.3852749 | 0.0008819 | 0.108795 | 0.4131182 | 0.04047262 |
| EBI3_IL27 | Q14213_Q8 | 0.2431614 | 0.790902 | 0.5477406 | 3.37300187 | 0.00091191 | 0.10090161 | 0.38542119 | 0.04047262 |
| PTGDS | P41222 | 0.23451401 | 0.916711 | 0.68219699 | 3.36958846 | 0.00092241 | 0.09717525 | 0.37185277 | 0.04047262 |
| MAMDC2 | Q7Z304 | 0.29543077 | 0.172124 | -0.1233068 | 3.36860632 | 0.00092591 | 0.12236417 | 0.46849737 | 0.04047262 |
| CDH2 | P19022 | 0.21329598 | 1.153802 | 0.94050602 | 3.35850865 | 0.00095058 | 0.0880049 | 0.33858707 | 0.04047262 |
| PKD2 | Q13563 | 0.36725857 | 1.59993 | 1.23267143 | 3.36203275 | 0.00095147 | 0.151657 | 0.58286015 | 0.04047262 |
| FLT1 | P17948 | 0.19627686 | 0.612931 | 0.41665414 | 3.35496195 | 0.0009707 | 0.08082479 | 0.31172894 | 0.04047262 |
| CDH3 | P22223 | 0.3430461 | 1.071107 | 0.7280609 | 3.35552715 | 0.00097342 | 0.14126301 | 0.54482919 | 0.04047262 |
| TNFRSF11A | Q9Y6Q6 | 0.3269918 | 1.053673 | 0.7266812 | 3.34951615 | 0.00097615 | 0.13442752 | 0.51955607 | 0.04047262 |
| VNN1 | O95497 | -0.4322726 | -0.64275 | -0.2104774 | -3.3498616 | 0.00098434 | -0.6868964 | -0.1776487 | 0.04047262 |
| LGALS9 | O00182 | 0.20358111 | 0.795339 | 0.59175789 | 3.33977476 | 0.00099523 | 0.08340252 | 0.32375969 | 0.04047262 |
| NECTIN4 | Q96NY8 | 0.29932466 | 0.85396 | 0.55463534 | 3.34730578 | 0.0010104 | 0.12276679 | 0.47588253 | 0.04047262 |
| HEG1 | Q9ULI3 | -0.1549192 | 0.101971 | 0.25689023 | -3.335411 | 0.00102049 | -0.2465247 | -0.0633138 | 0.04047262 |
| IL1RL1 | Q01638 | 0.47060336 | 1.177459 | 0.70685564 | 3.33485986 | 0.00103232 | 0.19218743 | 0.7490193 | 0.04047262 |
| CHRDL2 | Q6WN34 | 0.30564243 | 0.231371 | -0.0742714 | 3.32627142 | 0.00104588 | 0.12445861 | 0.48682625 | 0.04047262 |
| LMNB2 | Q03252 | 0.3117364 | 0.852777 | 0.5410406 | 3.33041362 | 0.00107038 | 0.12691971 | 0.49655309 | 0.04088304 |
| ADGRG1 | Q9Y653 | 0.47541438 | 1.120561 | 0.64514662 | 3.31630861 | 0.00109963 | 0.19257432 | 0.75825445 | 0.04146175 |
| TXNDC15 | Q96J42 | 0.17415514 | 0.543801 | 0.36964586 | 3.30382613 | 0.00113108 | 0.07020662 | 0.27810365 | 0.04210765 |
| SAP18 | O00422 | -0.2823998 | -0.358981 | -0.0765812 | -3.2940859 | 0.00116005 | -0.451407 | -0.1133926 | 0.04264645 |
| DMD | P11532 | 0.32565159 | 1.391314 | 1.06566241 | 3.28747036 | 0.00121568 | 0.13018606 | 0.52111713 | 0.04413955 |
| DSG2 | Q14126 | 0.16857202 | 0.777363 | 0.60879098 | 3.26759743 | 0.00127511 | 0.06684845 | 0.27029559 | 0.04573278 |
| CHCHD6 | Q9BRQ6 | 0.24396092 | 0.916294 | 0.67233308 | 3.25250432 | 0.00136786 | 0.09594641 | 0.39197543 | 0.04846838 |
| GRN | P28799 | 0.13813676 | 0.386133 | 0.24799624 | 3.23423314 | 0.00144125 | 0.05388186 | 0.22239166 | 0.0495752 |
| GPC1 | P35052 | 0.18668695 | 0.147808 | -0.0388789 | 3.22898884 | 0.00144924 | 0.07268905 | 0.30068484 | 0.0495752 |
| SHISA5 | Q8N114 | 0.26420713 | 1.021056 | 0.75684887 | 3.23603349 | 0.00146535 | 0.10300001 | 0.42541424 | 0.0495752 |
| C1QL2 | Q7Z5L3 | -0.2579222 | 0.225568 | 0.48349023 | -3.2277069 | 0.00146652 | -0.4155299 | -0.1003146 | 0.0495752 |
| TNFRSF8 | P28908 | 0.25348662 | 0.87994 | 0.62645338 | 3.21807168 | 0.00149221 | 0.0982176 | 0.40875563 | 0.0498705 |

# Table S11. Circulating cachexia mediator captured by Olink assay.

| GDF15^1^ | TNF^2^ | IL5^3^ | CCL11^4^ | CCL4^5^ | IL15^6^ | IL10^7^ | FGF2^8^ |
| --- | --- | --- | --- | --- | --- | --- | --- |
| IL1RN^9^ | CSF3^10^ | VEGFA^11^ | IL9^12^ | PDGFB^13^ | CXCL10^14^ | IL1B^15^ | IL7^16^ |
| IL6^15^ | CCL5^14^ | IL2^17^ | CCL2^18^ | IL4^19^ | IFNG^20^ | CSF2^21^ | IL13^21^ |

**Table S12. Technical information on local CT imaging analysis.**

| **Site** | **Instrument** | **Type of Imaging Study** | **Phase of contrast** | **Image thickness** | **Tube voltage** | **Tube current** |
| --- | --- | --- | --- | --- | --- | --- |
| I | GE Discovery STE | Whole body FDG PET-CT |  | 2.5 | 120 | 181 |
| I | Philips Brilliance 64 | CT-Abdomen with contrast | Late arterial/arterial phase | 3 | 120 | 105 |
| II | Toshiba Aquilion | CT-Abdomen with contrast | Late arterial/arterial phase | 1 | 120 | 103 |
| II | GE Discovery STE | Whole body FDG PET-CT |  | 3.75 | 140 | 80 |
| III | Philips Brilliance 64 | CT-Abdomen with contrast | Late arterial/arterial phase | 3 | 100 | 203 |
| III | GE Discovery STE | Whole body FDG PET-CT |  | 2.5 | 120 | 181 |
| IV | GE Discovery STE | Whole body FDG PET-CT |  | 3.75 | 120 | 43 |
| IV | GE Optima CT660 | CT-Abdomen with contrast | Late arterial/arterial phase | 0.625 | 120 | 121 |
| V | GE Discovery 690 | Whole body FDG PET-CT |  | 2.5 | 140 | 199 |
| VI | GE Discovery 710 | Whole body FDG PET-CT |  | 2.5 | 120 | 62 |
| VI | GE Revolution CT | CT-Abdomen with contrast | Late arterial/arterial phase | 1.25 | 120 | 282 |
| VII | Siemens Biograph 6 | Whole body FDG PET-CT |  | 5 | 130 | 75 |
| VII | Toshiba Aquilion | CT-Abdomen with contrast | Late arterial/arterial phase | 1 | 120 | 97 |
| VIII | Toshiba Aquilion | CT-Abdomen with contrast | Late arterial/arterial phase | 3 | 120 | 216 |
| VIII | Siemens Biograph 6 | Whole body FDG PET-CT |  | 3 | 120 | 140 |
| IX | Siemens Somatom Def AS | Whole body FDG PET-CT |  | 3 | 120 | 95 |
| IX | GE LightSpeed VCT | CT-Abdomen with contrast | Late arterial/arterial phase | 1.25 | 100 | 428 |
| X | Siemens Biograph 6 | Whole body FDG PET-CT |  | 5 | 130 | 75 |
| X | GE Revolution GSI | CT-Abdomen with contrast | Late arterial/arterial phase | 1.25 | 120 | 100 |
| XI | Philips Ingenuity Core 128 | CT-Abdomen with contrast | Late arterial/arterial phase | 1 | 120 | 268 |
| XI | Siemens Biograph 64 | Whole body FDG PET-CT |  | 3 | 120 | 88 |
| XII | GE Discovery 690 | Whole body FDG PET-CT |  | 2.5 | 120 | 51 |
| XII | Siemens SomatomDef Flash | CT-Abdomen with contrast | Late arterial/arterial phase | 2 | 120 | 74 |
| XIII | Siemens Somatom Def AS | Whole body FDG PET-CT |  | 3 | 100 | 156 |
| XIII | Siemens Sensation 64 | CT-Abdomen with contrast | Late arterial/arterial phase | 1.5 | 120 | 403 |
| XIV | GE Discovery STE | Whole body FDG PET-CT |  | 2.5 | 120 | 43 |
| XIV | Philips Brilliance 64 | CT-Abdomen with contrast | Late arterial/arterial phase | 3 | 100 | 156 |
| XV | GE Discovery CT750 HD | CT-Abdomen with contrast | Late arterial/arterial phase | 0.625 | 129 | 161 |
| XV | GE Discovery ST | Whole body FDG PET-CT |  | 3.75 | 140 | 80 |

# Table S13. Body composition precision metrics.

According to Arribas et al, 2022 approach. Precision error and least significant change (LSC) of the cross-sectional areas (cm^2^) for skeletal muscle (SKM), subcutaneous adipose tissue (SAT) and visceral adipose tissue (VAT), derived from two independent DAFS runs on a random subset of 50 CT scans from TRACERx.

RMS SD, root-mean-square error standard deviation; %CV, % coefficient of variability; LSC, least significant change; LSC _RMS SD_, least significant change for the root-mean -square deviation; LSC _%CV_, least significant change for the root-mean -square percent coefficient of variation.

|  | **RMS SD** | **%CV** | **LSC_RMS SD_** | **LSC_%CV_** |
| --- | --- | --- | --- | --- |
| **SKM** | 0.001325825 | 0.002426404 | 0.003672536 | 0.006721138 |
| **SAT** | 0.010020072 | 0.010020072 | 0.027755599 | 0.027755599 |
| **VAT** | 0.000937999 | 0.009108276 | 0.002598257 | 0.025229926 |

**SUPPLEMENTARY FIGURES**
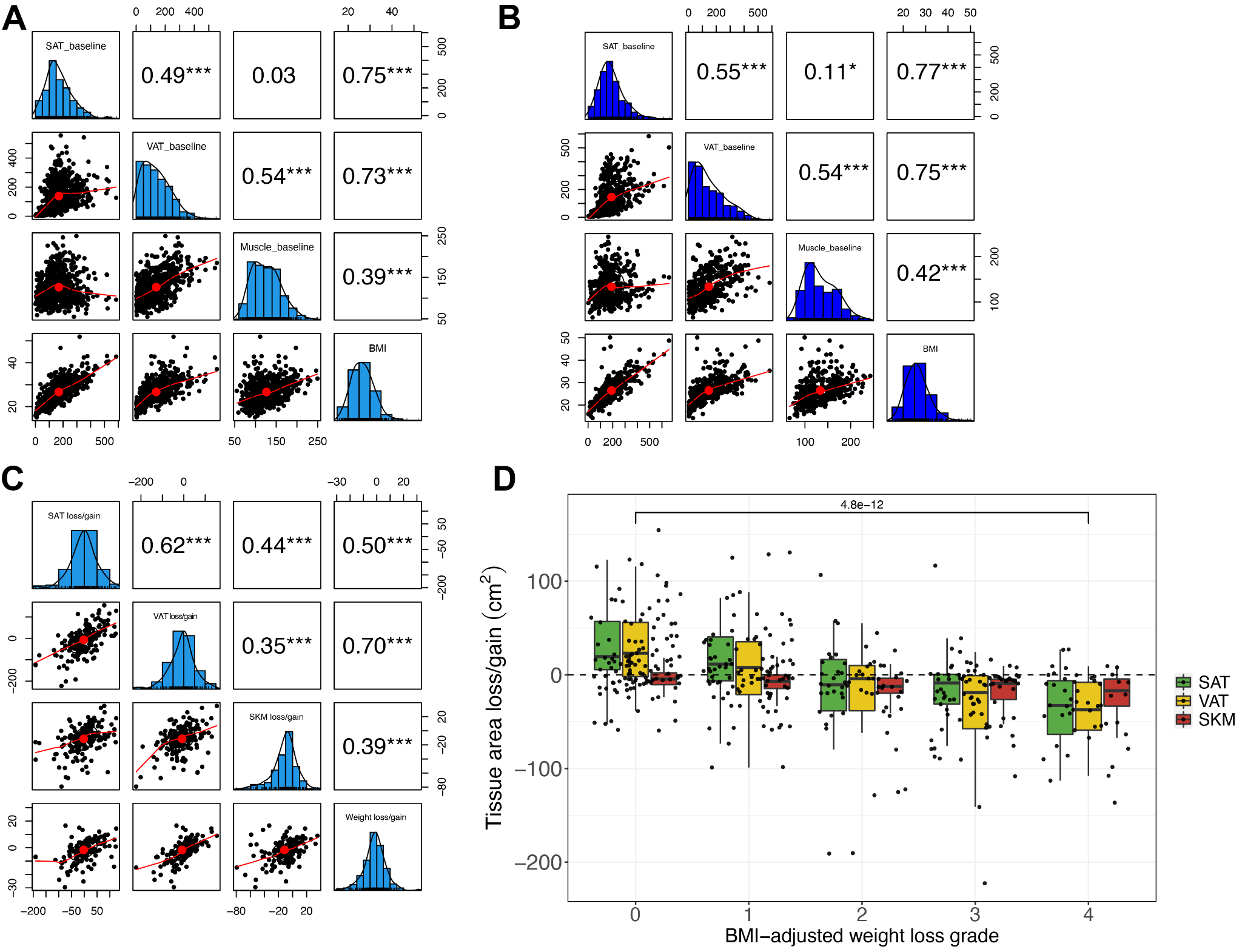


# Figure S1. Correlation between body composition metrics and body weight.

**A** Spearman correlation in the TRACERx cohort between subcutaneous adipose tissue (SAT), visceral adipose tissue (VAT), skeletal muscle (SKM) and body mass index (BMI) at primary diagnosis. **B** Spearman correlation in the BLCS cohort between subcutaneous adipose tissue (SAT), visceral adipose tissue (VAT), skeletal muscle (SKM) and body mass index (BMI) at primary diagnosis **C** Spearman correlation between loss/gain of SAT, VAT, SKM and body weight between primary diagnosis and first relapse. **D** Losses and gains in cm^2^ of SAT (green), VAT (yellow) and SKM (red) according to BMI-adjusted weight loss grade 0 to 4. Bracket indicates p-value from two-sided Wilcoxon test; box plots represent lower quartile, median and upper quartile, whiskers extend to a maximum of 1.5 × IQR beyond the box. Points indicate individual data points. *** indicates p-value <0.001.


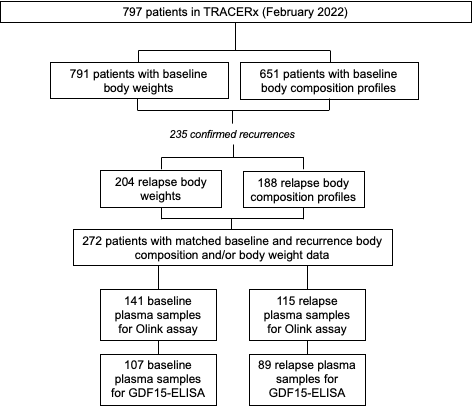


# Figure S2. Patient and sample selection in TRACERx.

**
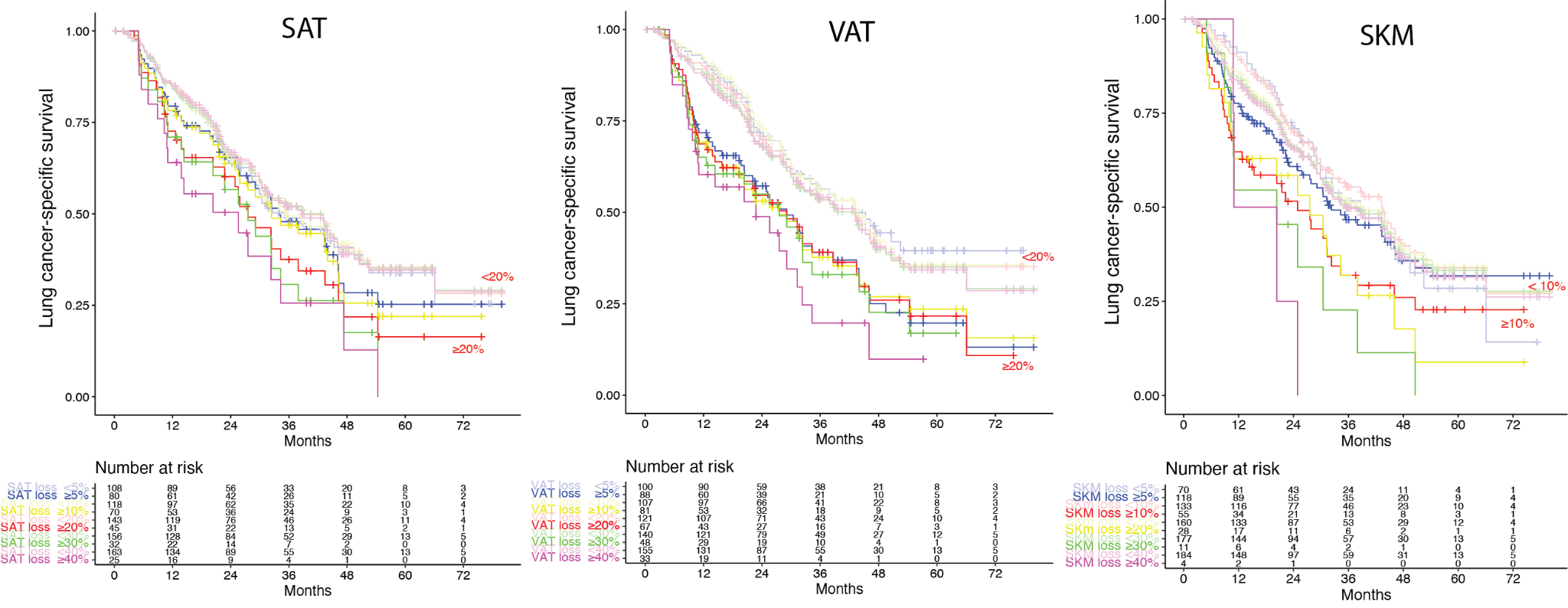
**

# Figure S3. Identification of prognostically relevant threshold of SAT, VAT and SKM loss.

Thresholds from 5% to 40% were explored to identify clinically relevant groups of patients with short lung cancer-specific survival according to loss of subcutaneous adipose tissue (SAT), visceral adipose tissue (VAT) and skeletal muscle tissue (SKM).

**
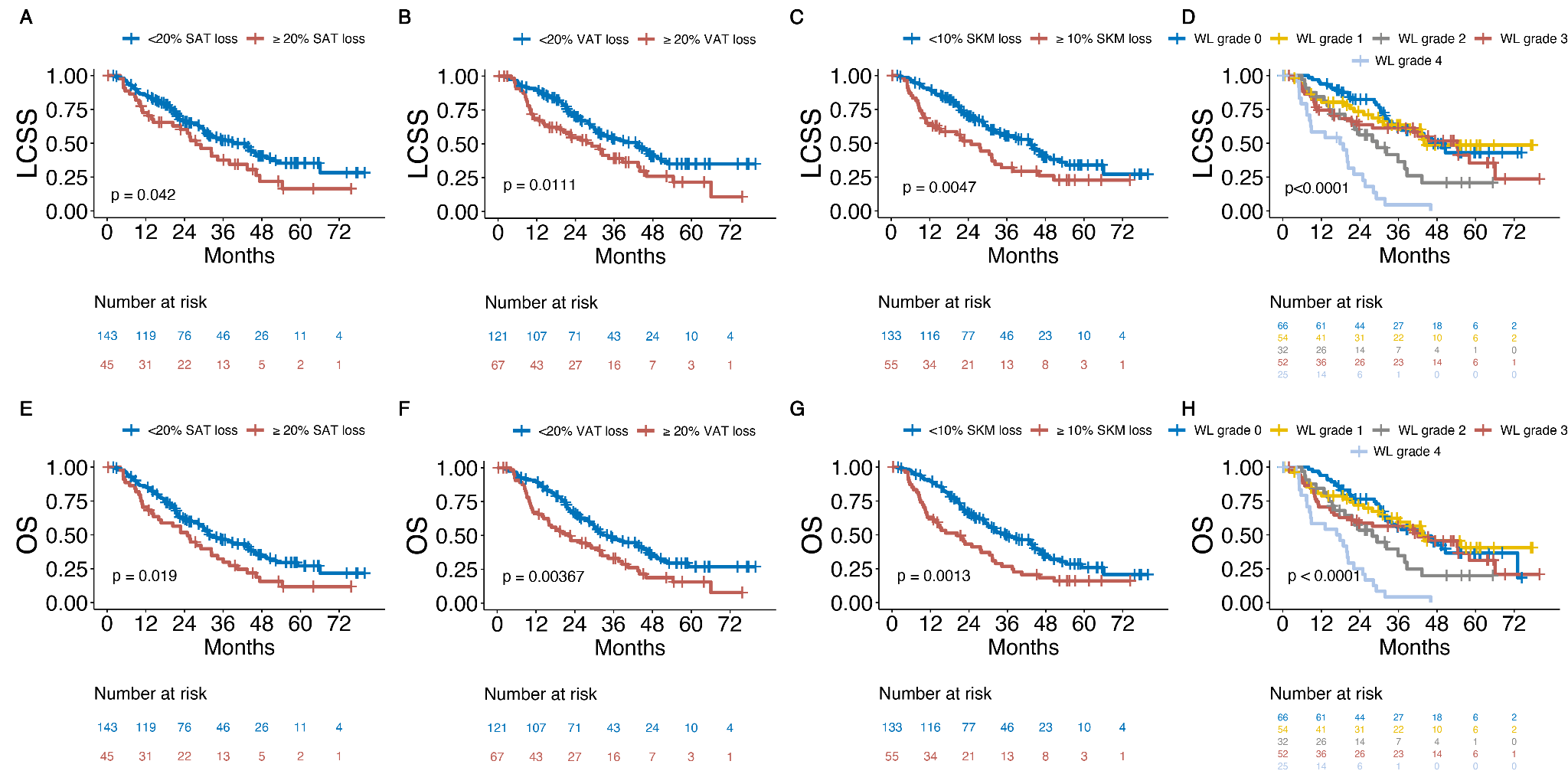
**

# Figure S4. Survival according to SAT, VAT, SKM and BMI-adjusted weight loss.

**A, B, C, D** Kaplan-Meier analysis of lung cancer-specific survival (LCSS) according to </≥20% SAT loss, </≥20% VAT loss, </≥10% skeletal muscle (SKM) loss, BMI-adjusted weight loss grade (WL grade) 0-4. **E, F, G, H** Kaplan-Meier analysis of overall survival (OS) according to </≥20% SAT loss, </≥20% VAT loss, </≥10% skeletal muscle (SKM) loss, BMI-adjusted weight loss grade (WL grade) 0-4. P-values from Cox regression model.

**
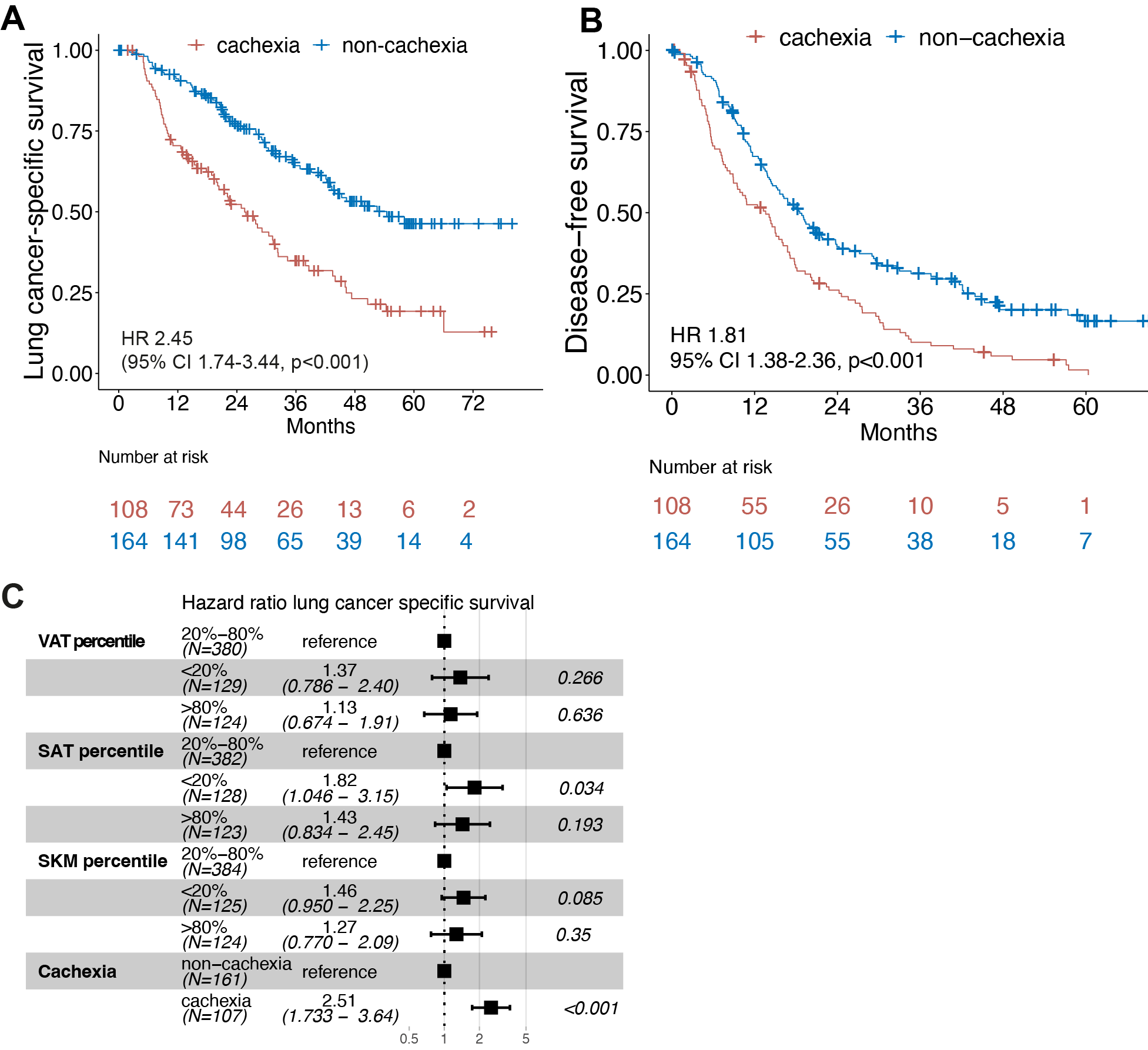
**

# Figure S5. Lung cancer-specific survival and disease-free survival according to cachexia status. A LCSS according to cachexia (red) and non-cachexia (blue) groups. B DFS according to cachexia and non-cachexia group. P-values from Cox regression model. C Multivariable analysis of lung cancer-specific survival, adjusted for age, sex, BMI, smoking status, disease stage, histological subtype, ethnicity, and adjuvant therapy.

#
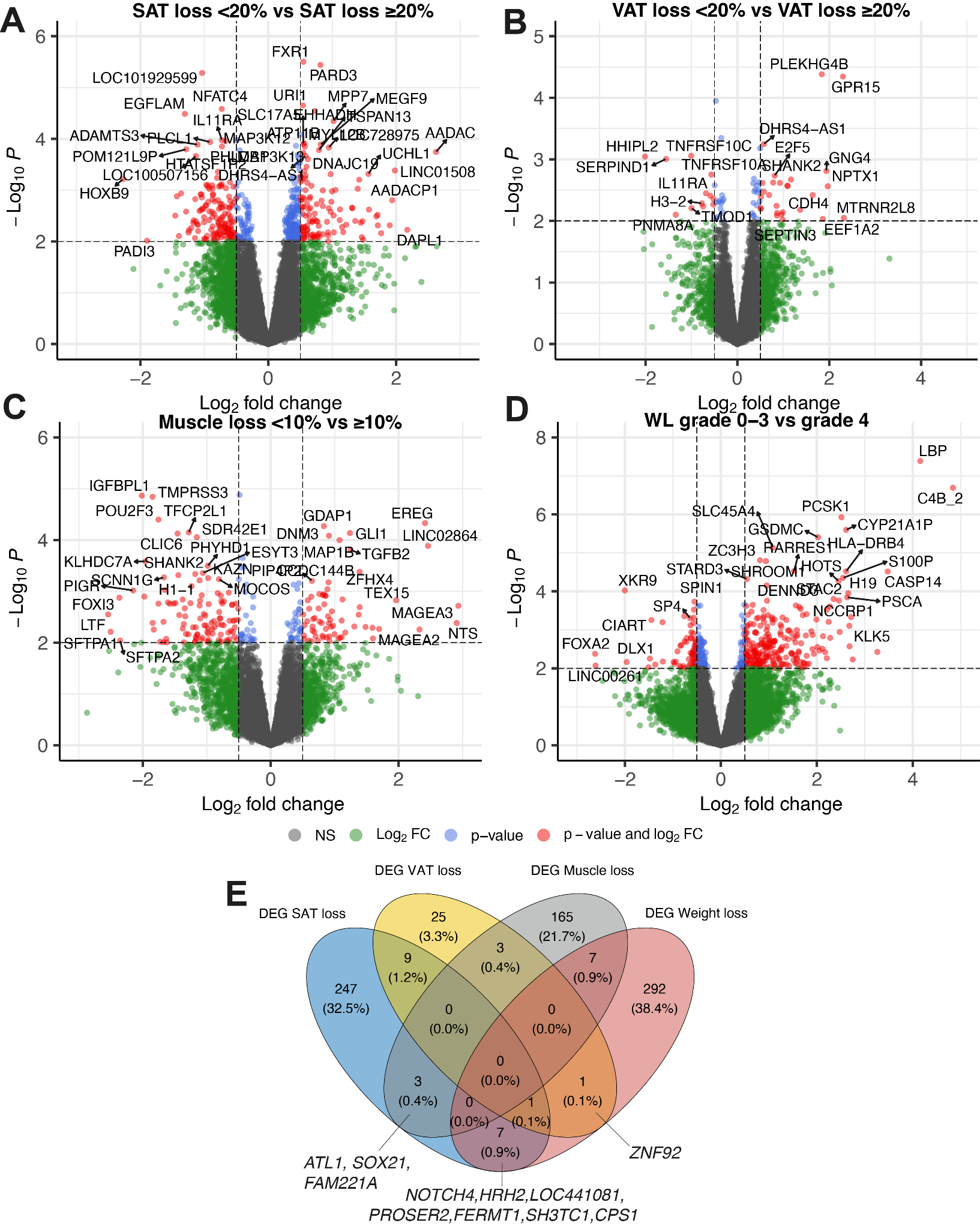


# Figure S6. Differential gene expression according to SAT, VAT, SKM and body weight loss.

Tumour differential gene expression between patients with **A** SAT loss <20% versus ≥20%, **B** VAT loss <20% versus ≥20%, **C** Muscle loss <10% versus ≥10%, **D** BMI adjusted weight loss grade 0-3 versus 4, all adjusted for number of tumour regions, sex, and histology. **E** Overlap of differentially expressed genes (DEG) between the ≥20% SAT, ≥20% VAT, ≥10% SKM and grade 4 weight loss groups.

**
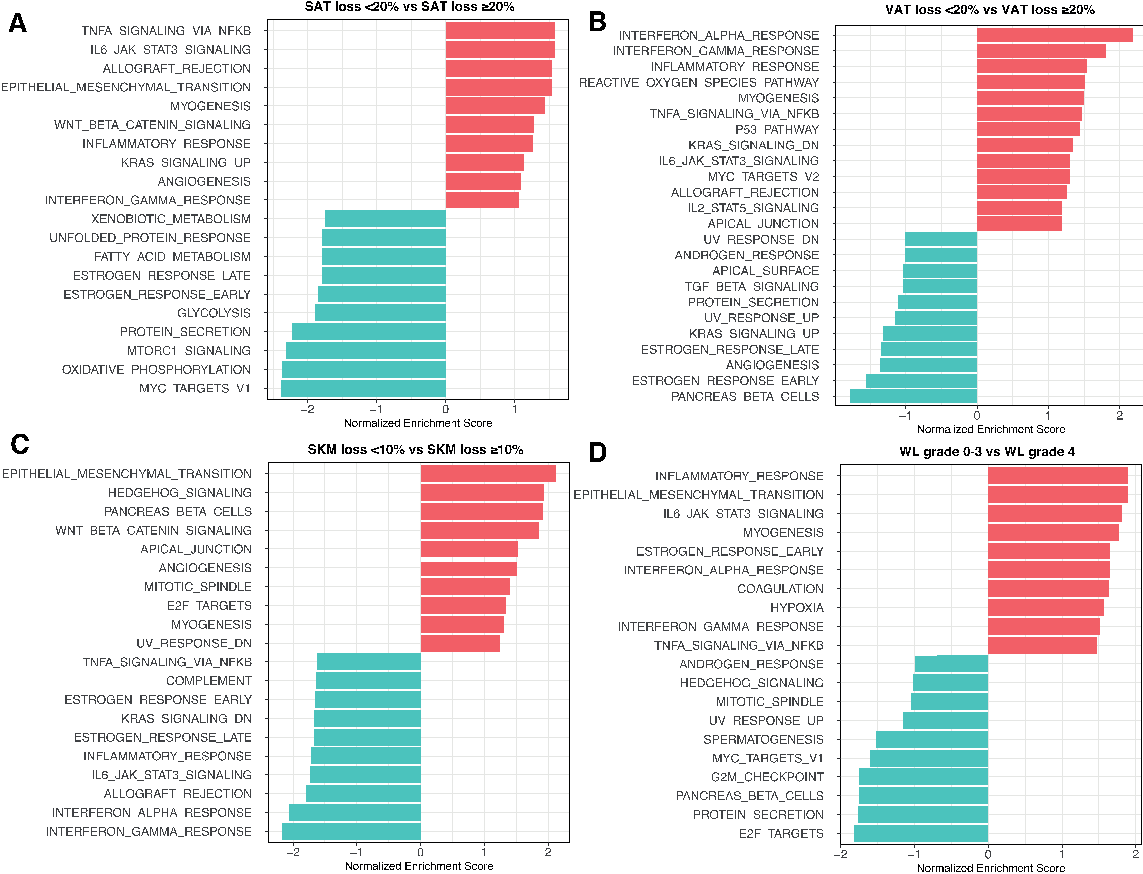
**

# Figure S7. Gene set enrichment according to SAT, VAT, SKM and body weight loss.

Red bars indicate enrichment in patients with higher loss of tissue/body weight, green bars indicate enrichment in patients with lower loss of tissue/body weight. All differential comparisons adjusted for histology and sex.

**
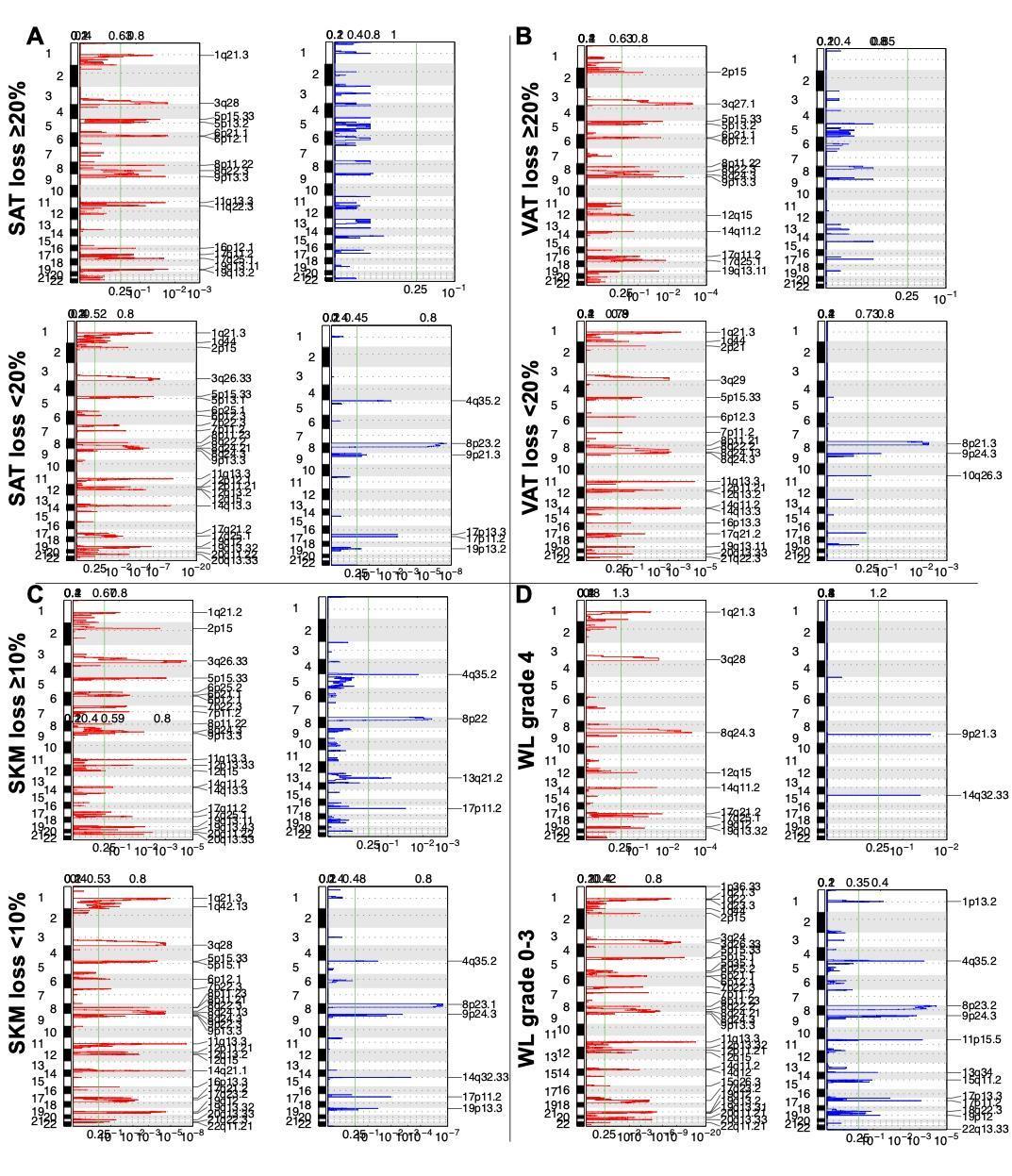
**

# Figure S8. GISTIC copy number profiles according to SAT, VAT, SKM and body weight loss.

**A** subcutaneous adipose tissue (SAT) loss, **B** visceral adipose tissue (VAT) loss, **C n** skeletal muscle tissue (SKM) loss, **D** BMI-adjusted weight loss (WL) grade. Y-axes indicate chromosomal positions (1-22), red plots indicate gains, blue plots indicate losses. X-axes indicate q-values. Most significant peaks are indicated on the right of each panel; regions with FDR q≤0.25 (vertical green line) are considered significant. GISTIC G-Scores are plotted on top of each panel.


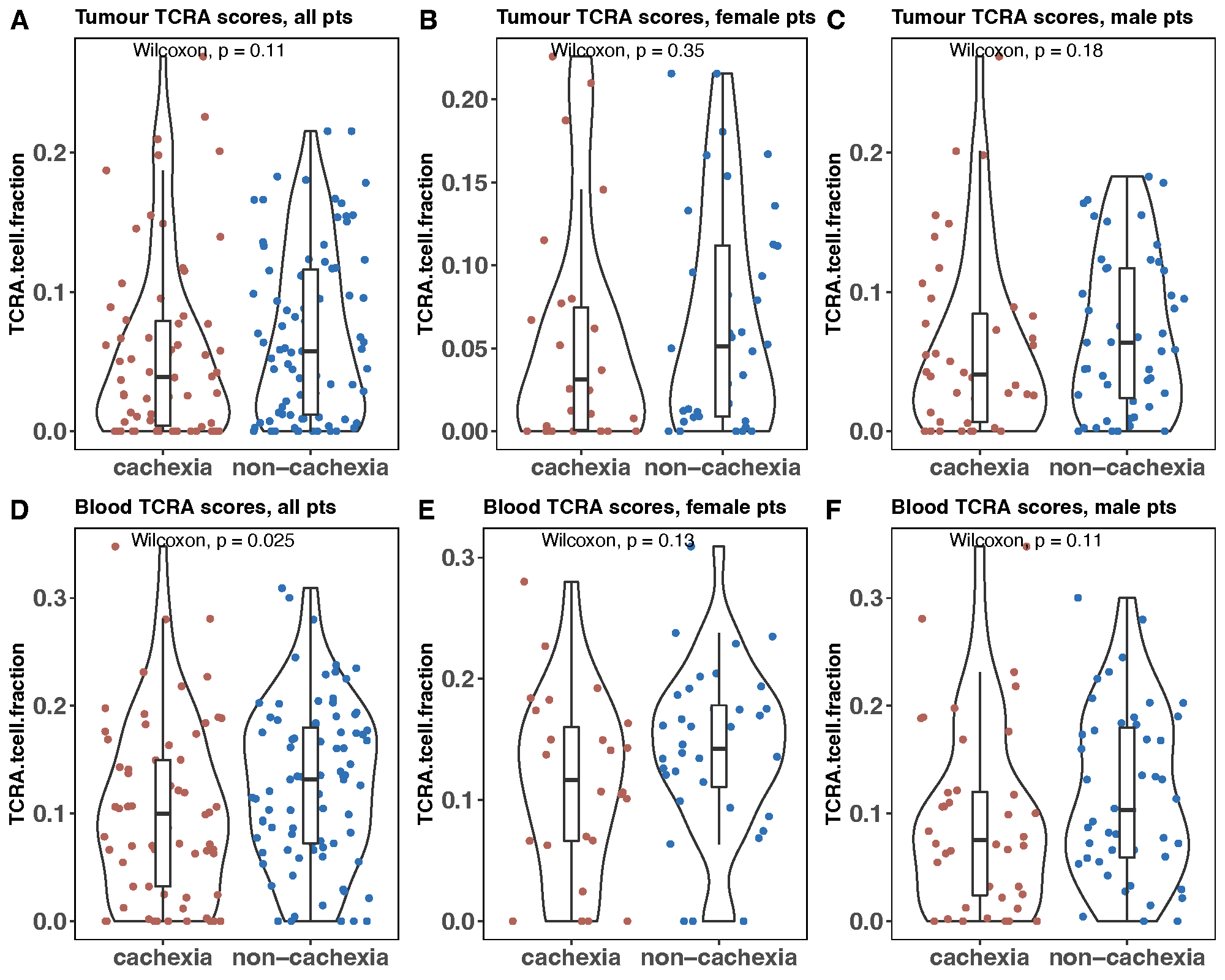


# Figure S9. TCRA scores according to CAC and non-CAC groups.

**A** Tumour TCRA scores according to CAC and non-CAC status. **B** Tumour TCRA scores according to CAC and non-CAC in male and **C** female patients. **D** Blood TCRA scores according to CAC and non-CAC status. **E** Blood TCRA scores in blood in male and **F** female patients.


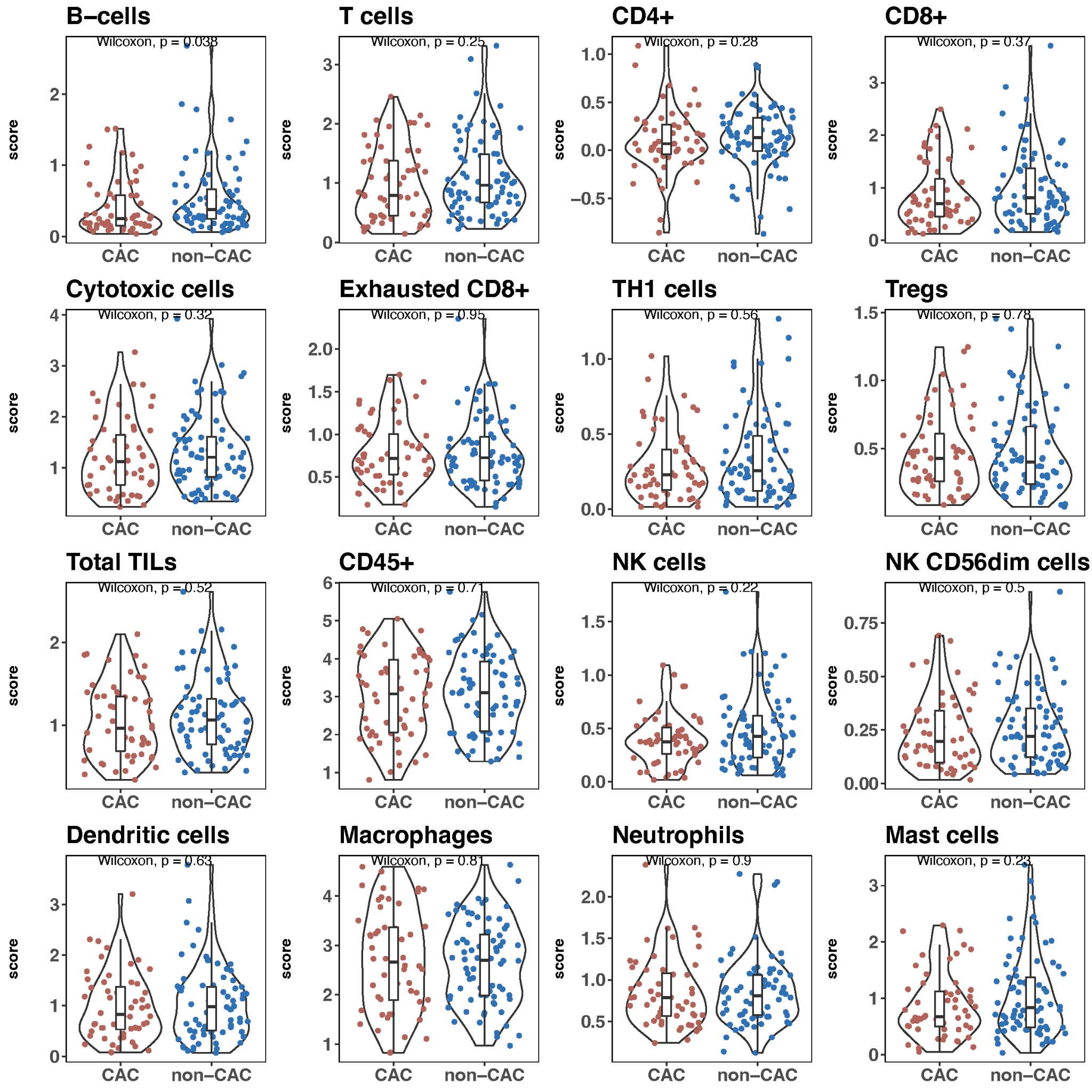


# Figure S10. Danaher scores according to CAC and non-CAC groups.

No significant difference was observed between cachexia and non-cachexia groups after Bonferroni correction.

**
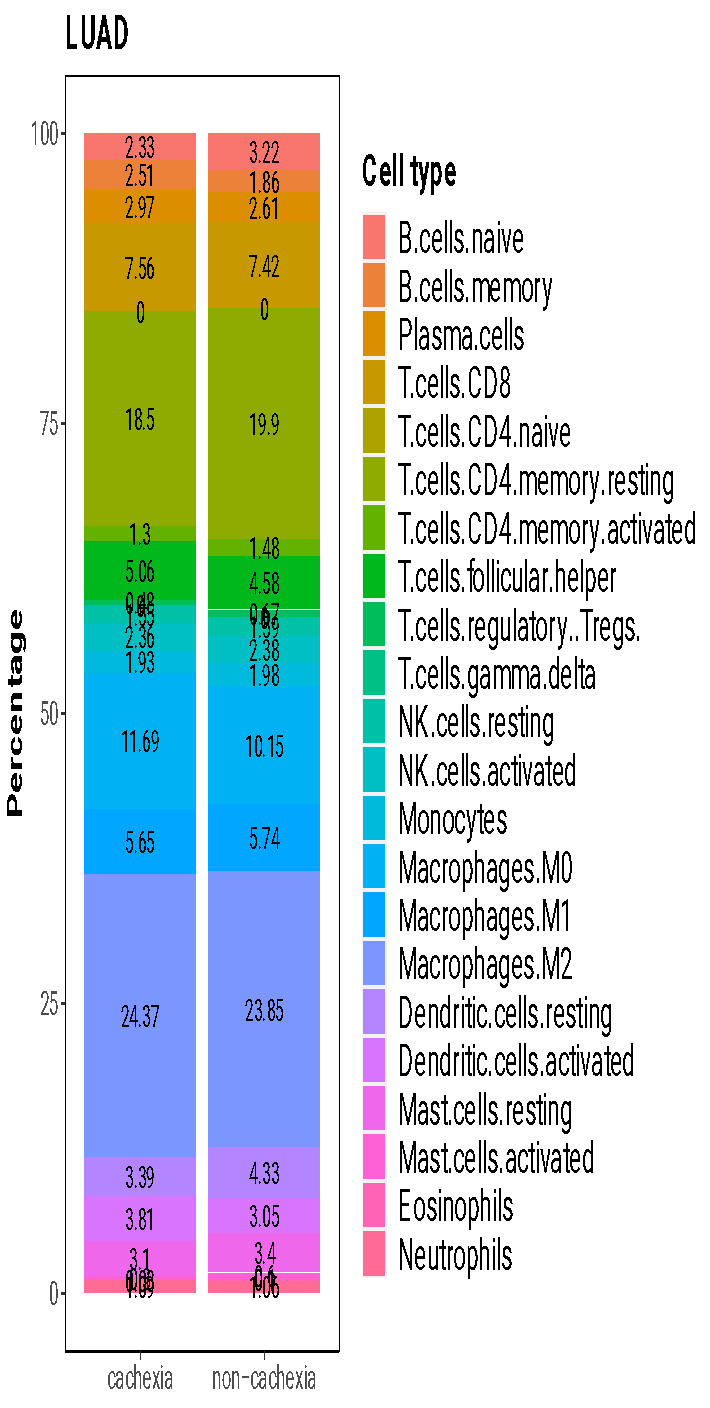

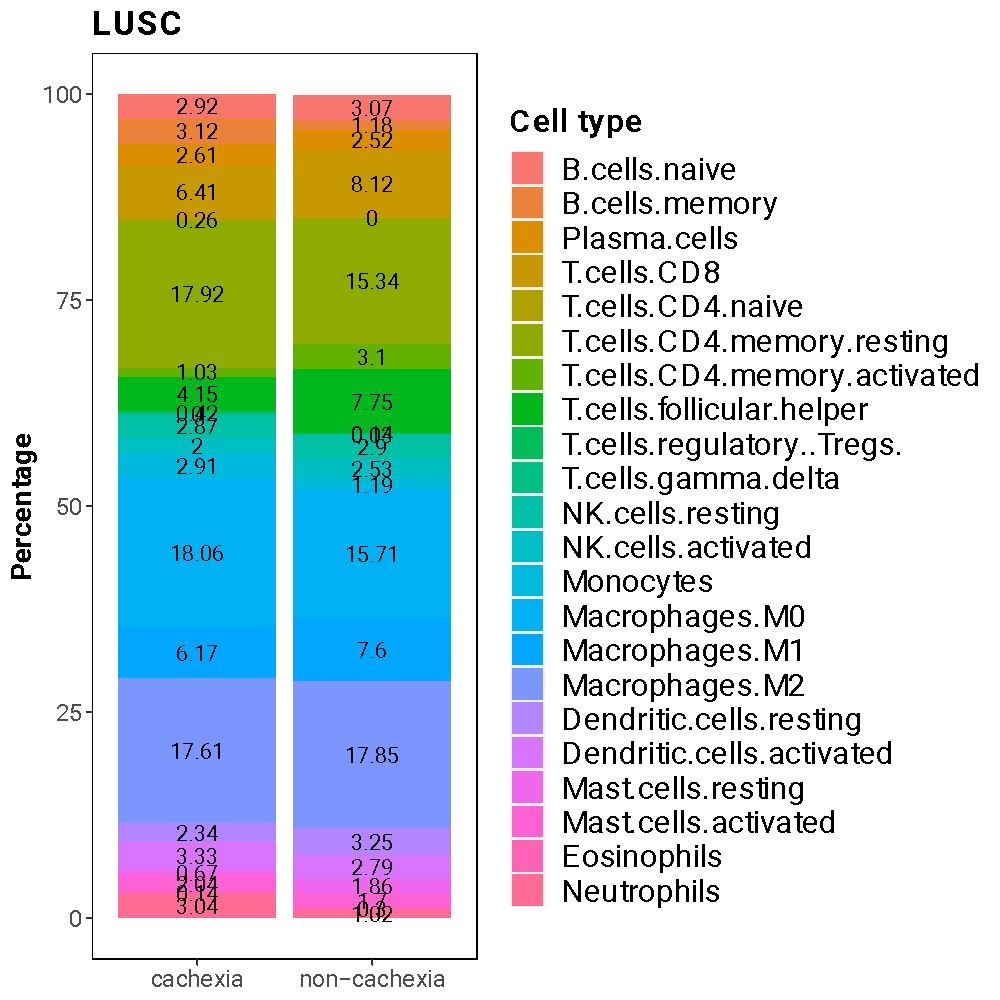
**

# Figure S11. Cell type deconvolution by CIBERSORTx according to CAC and non-CAC groups.

Y-axes indicate infiltration percentage, x-axes indicate cachexia or non-cachexia status, according to lung adenocarcinoma (LUAD) histology or lung squamous cell carcinoma (LUSC) histology.

#


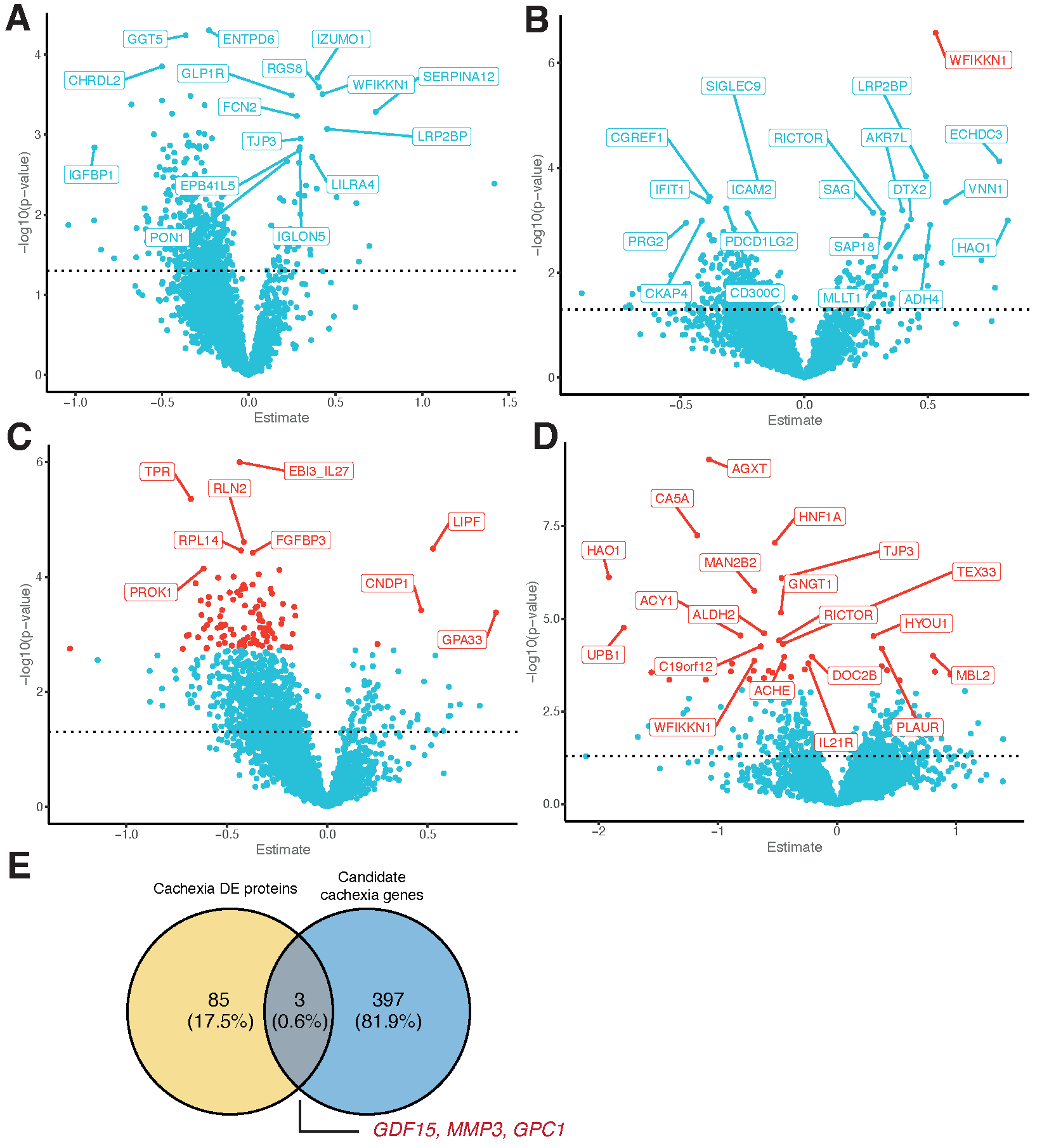


# Figure S12. Differential plasma protein expression.

**A** According to SAT loss. **B** VAT loss. **C** SKM loss. **D** Body weight loss. High estimates indicate higher levels, lower estimates indicate lower levels in patients with SAT/VAT/SKM/body weight loss. Red annotation indicates significant values according to paired t-test with Benjamin-Hochberg correction. **E** Overlaps between differentially expressed plasma proteins in cachexia patients and candidate cachexia genes.


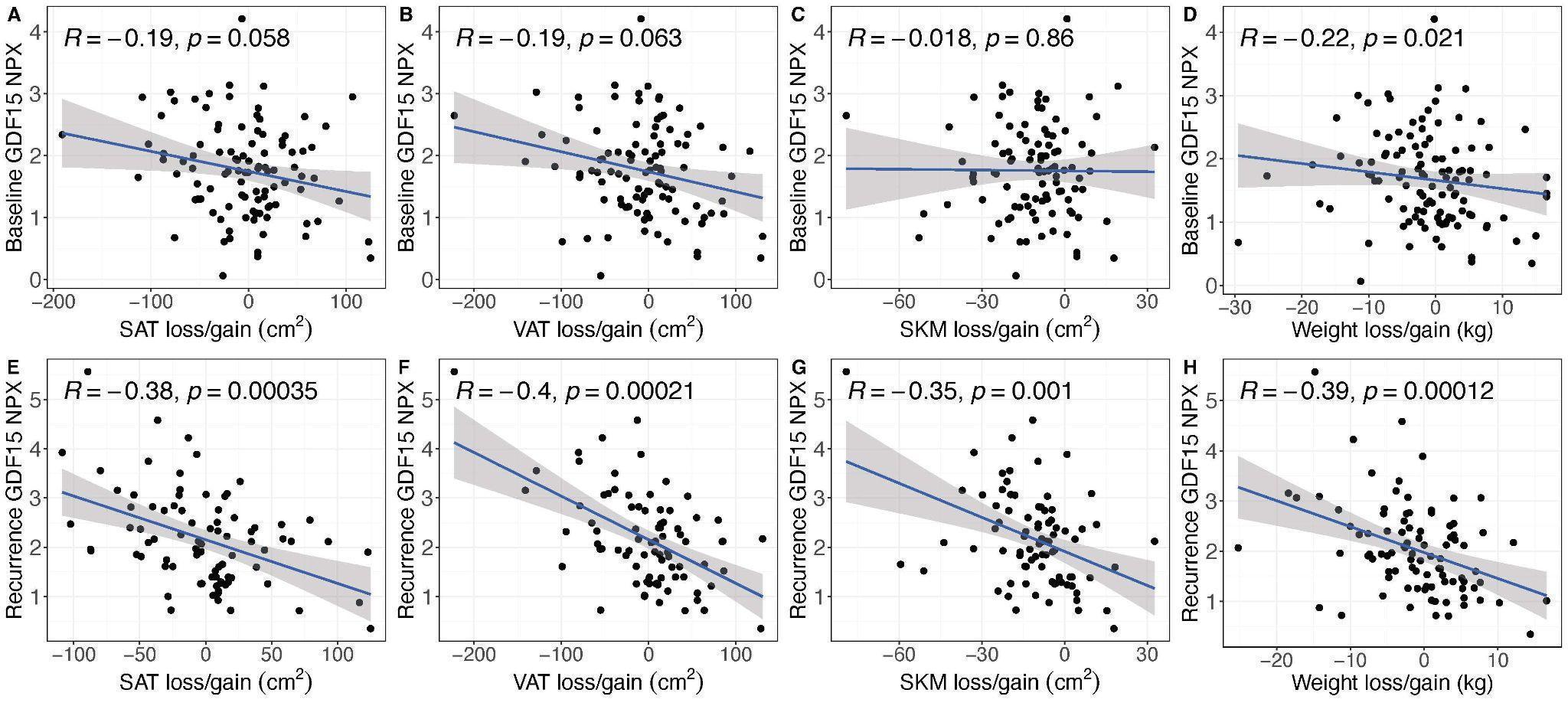


# Figure S13. Correlation between normalized plasma GDF15 expression (NPX) and changes in body composition and body weight.

Spearman correlation between baseline/recurrence plasma GDF15 NPX and changes in **A**/**E** subcutaneous adipose tissue (SAT), **B/F** visceral adipose tissue (VAT), **C/G** skeletal muscle tissue (SKM), body weight. Shaded areas indicate 95% confidence intervals.


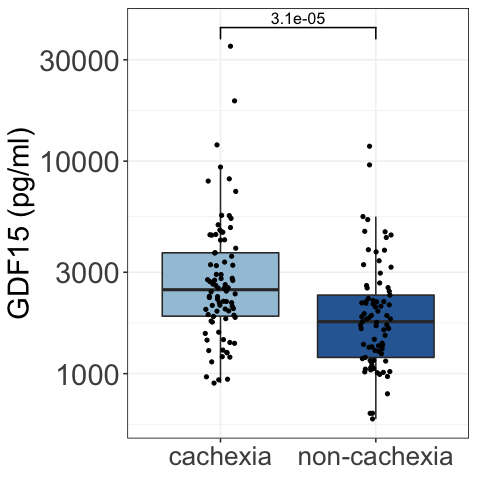


# Figure S14. Plasma GDF15 levels in patients with versus without cachexia.

Two-sided Wilcoxon test. Box plots represent lower quartile, median and upper quartile, whiskers extend to a maximum of 1.5 × IQR beyond the box. Points indicate individual data points. Y-axis represents log10 scales.


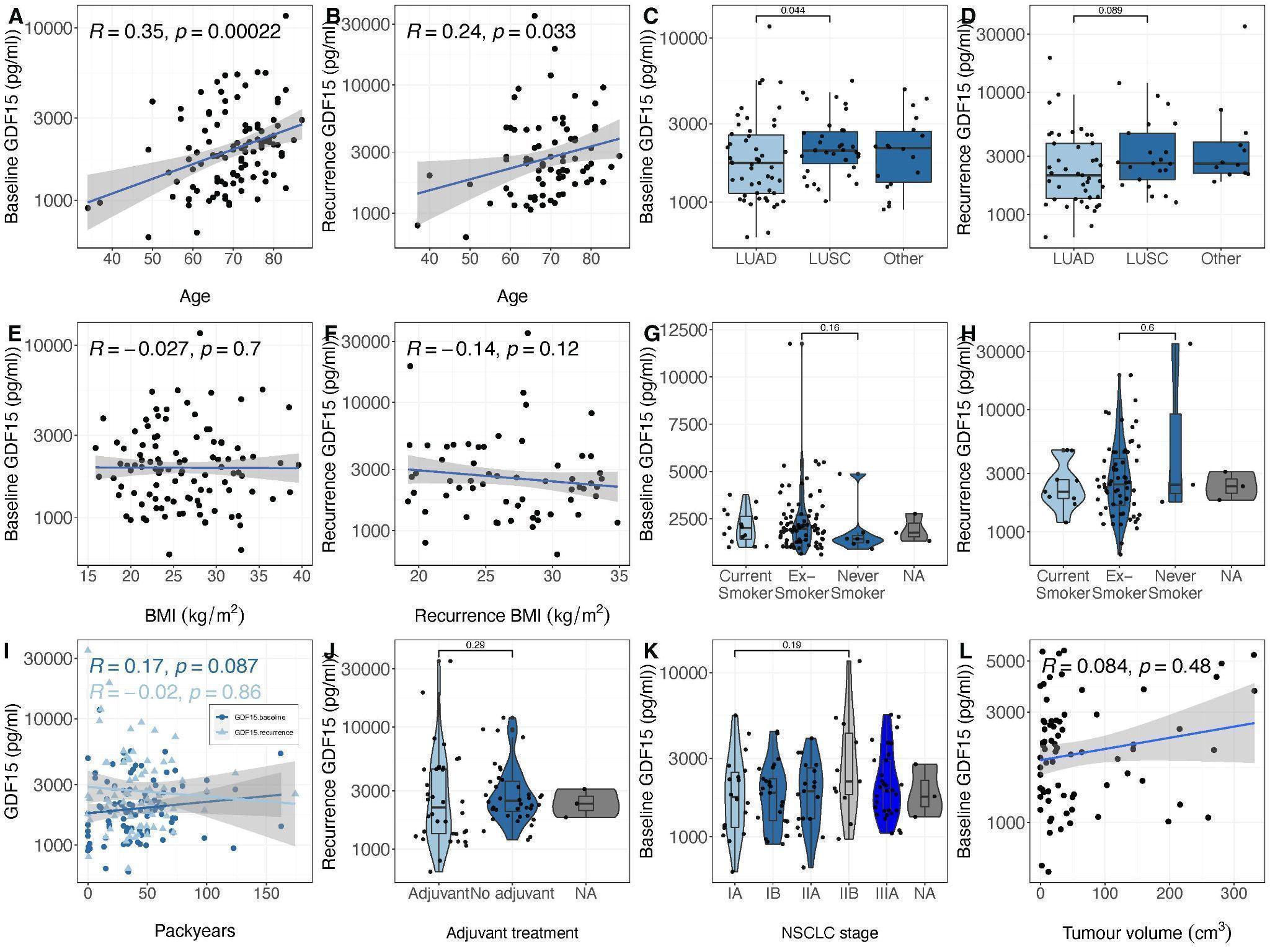


# Figure S15. Correlation of GDF15 plasma levels with clinical features.

Correlation between serum GDF15 and age (A, B), histology (C,D), body mass index (BMI) (D) and body mass index (BMI) (E,F), smoker status (G,H), packyears (I), adjuvant treatment (J), tumour stage (K) and tumour volume (L). NSCLC, non-small cell lung cancer; LUAD, lung adenocarcinoma; LUSC, lung squamous cell carcinoma.


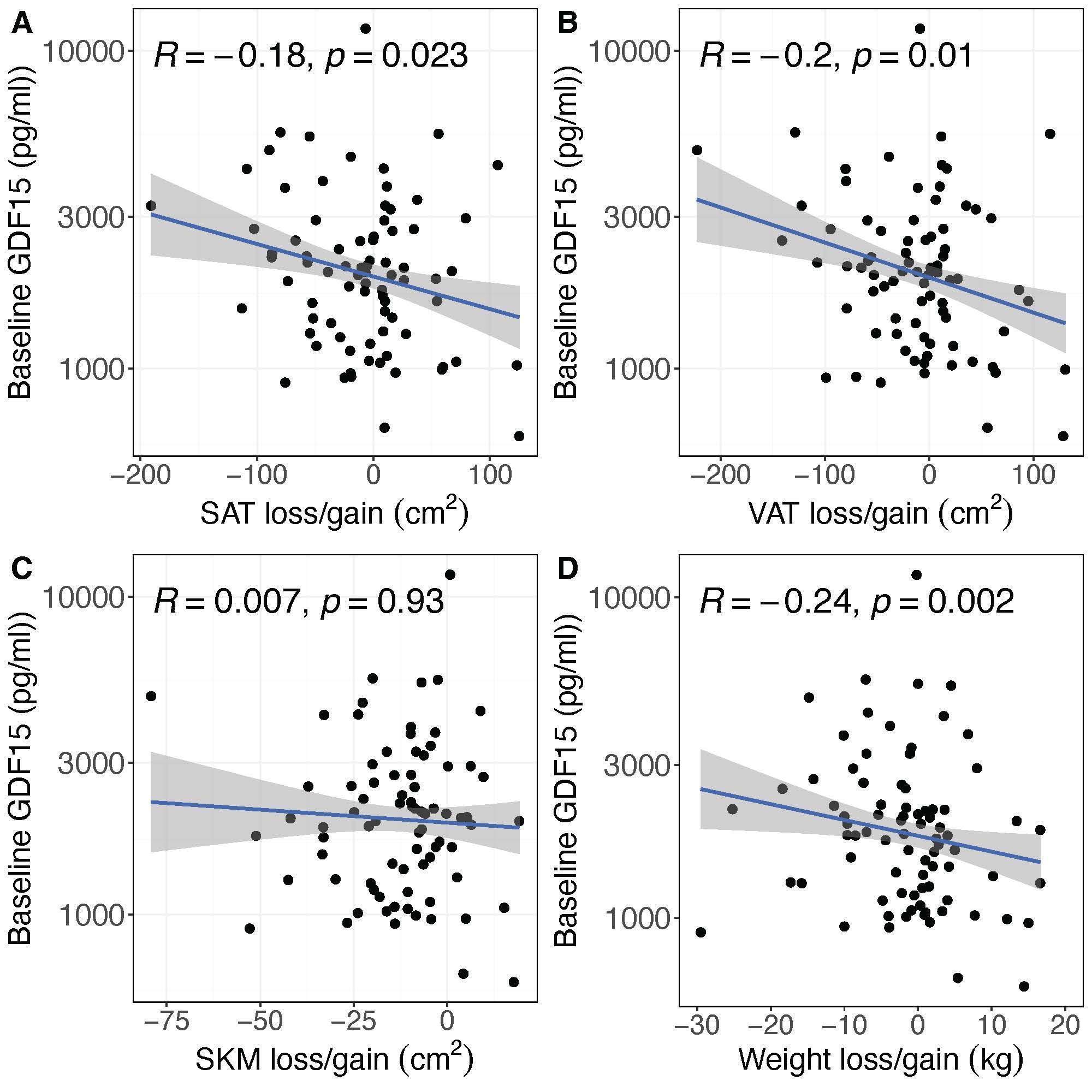


# Figure S16. Baseline plasma GDF15 levels and body composition/weight changes.

Spearman correlation between baseline plasma GDF15 levels and **A** subcutaneous adipose tissue, **B** visceral adipose tissue, **C** skeletal muscle change and **D** weight changes. Grey shade areas represent 95% confidence intervals. Y-axis represents log10 scales.


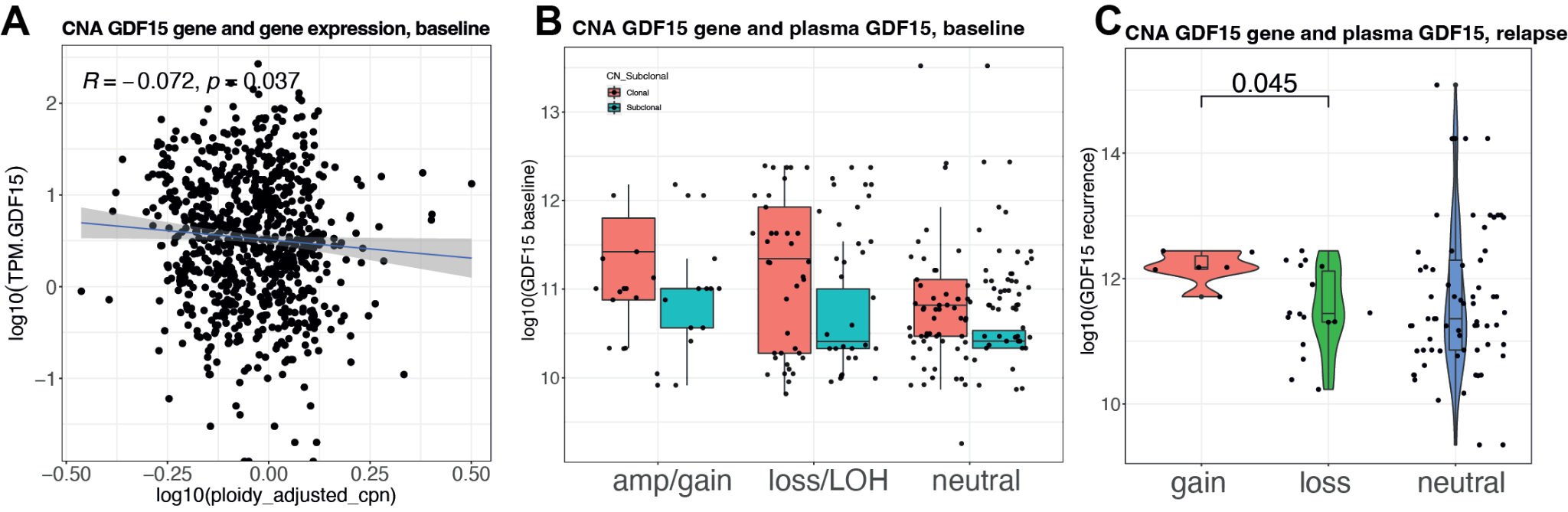


# Figure S17. Copy number alterations of GDF15.

**A** Ploidy-adjusted copy number compared to transcript-per-million GDF15 gene expression, at baseline, log10 transformed, Spearman’s correlation. N=245 **B** Clonal and subclonal copy number events in relation to circulating GDF15 levels, at baseline, log10 transformed. **C** Copy number events in relation to circulating GDF15 levels, at relapse, log10 transformed. Two-sided Wilcoxon test.


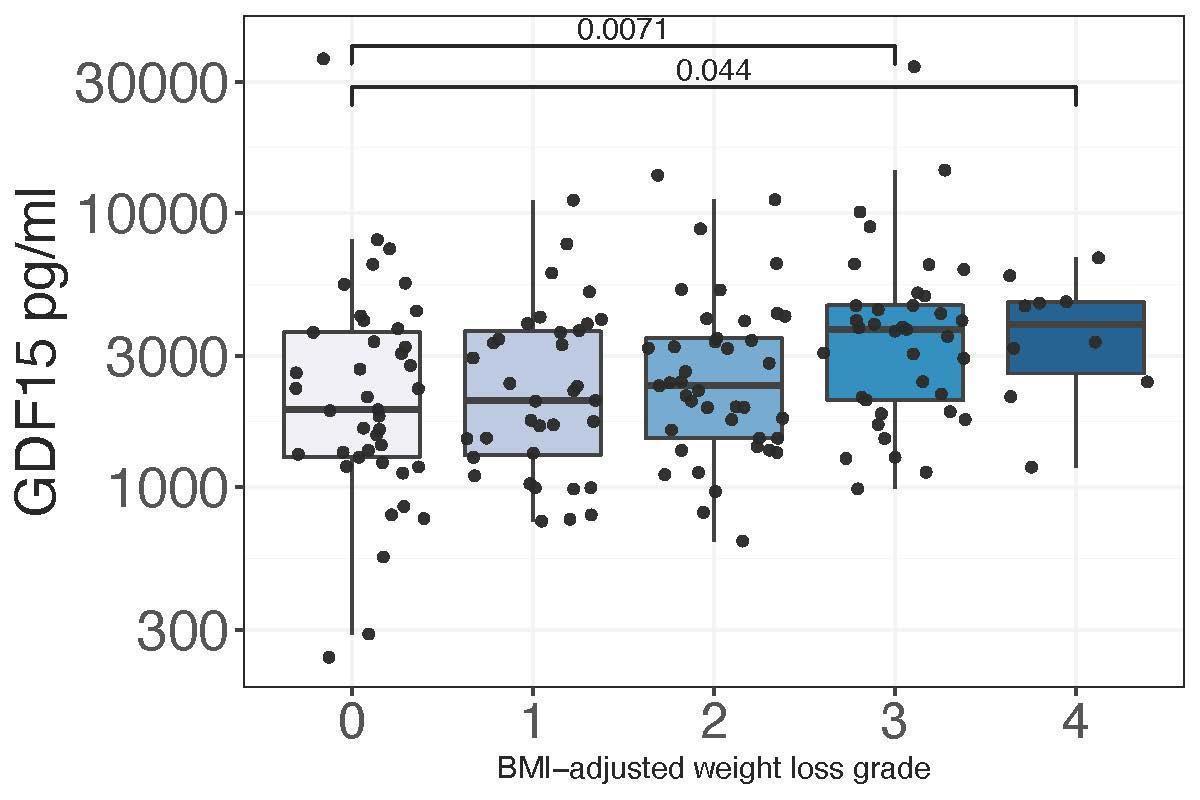


# Figure S18. Plasma GDF15 levels and BMI-adjusted weightloss in the ARCHER1009 cohort.

GDF15 levels according to BMI-adjusted weight change category in patients treated in the ARCHER1009 trial (two-sided wilcoxon test, error bars indicate standard deviation). Y-axis represents log10 scales.

*
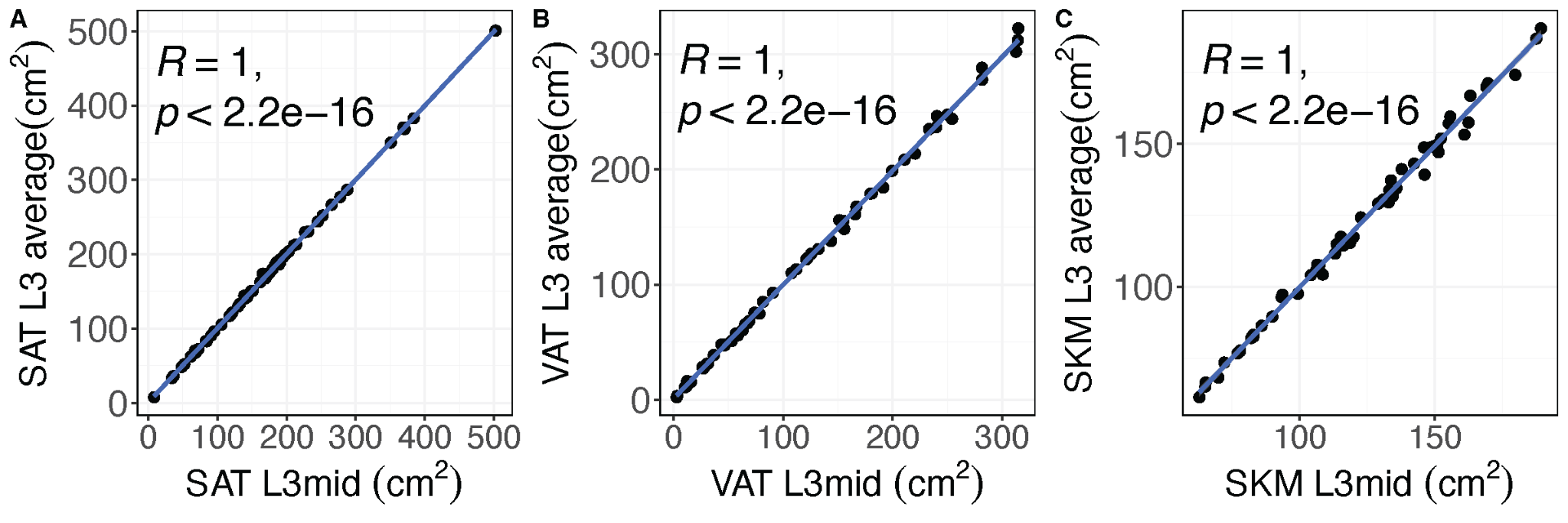
*

# Figure S19. Correlation between mid L3 and average L3 body composition measurements.

Spearman’s correlation between body composition measurements using L3mid (x-axis) and avg-L3mid[3] (y-axis) function. SAT, Subcutaneous adipose tissue (A); VAT, Visceral adipose tissue (B); SKM, Skeletal muscle tissue (C).

**SUPPLEMENTARY REFERENCES**

1. Speliotes, E.K.*, et al.* Association analyses of 249,796 individuals reveal 18 new loci associated with body mass index. *Nature genetics* **42**, 937-948 (2010).

2. Locke, A.E.*, et al.* Genetic studies of body mass index yield new insights for obesity biology. *Nature* **518**, 197-206 (2015).

3. Wen, W.*, et al.* Meta-analysis of genome-wide association studies in East Asian-ancestry populations identifies four new loci for body mass index. *Hum Mol Genet* **23**, 5492-5504 (2014).

4. Akiyama, M.*, et al.* Genome-wide association study identifies 112 new loci for body mass index in the Japanese population. *Nature genetics* **49**, 1458-1467 (2017).

5. Winkler, T.W.*, et al.* The Influence of Age and Sex on Genetic Associations with Adult Body Size and Shape: A Large-Scale Genome-Wide Interaction Study. *PLoS Genet* **11**, e1005378 (2015).

6. Johns, N.*, et al.* New genetic signatures associated with cancer cachexia as defined by low skeletal muscle index and weight loss. *J Cachexia Sarcopenia Muscle* **8**, 122-130 (2017).

7. Solheim, T.S.*, et al.* Is there a genetic cause for cancer cachexia? - a clinical validation study in 1797 patients. *Br J Cancer* **105**, 1244-1251 (2011).

8. Baranski, T.J.*, et al.* A high throughput, functional screen of human Body Mass Index GWAS loci using tissue-specific RNAi Drosophila melanogaster crosses. *PLOS Genetics* **14**, e1007222 (2018).

9. Lodge, W.*, et al.* Tumor-derived MMPs regulate cachexia in a Drosophila cancer model. *Dev Cell* **56**, 2664-2680.e2666 (2021).

10. Ding, G.*, et al.* Coordination of tumor growth and host wasting by tumor-derived Upd3. *Cell Rep* **36**, 109553 (2021).

11. Kwon, Y.*, et al.* Systemic organ wasting induced by localized expression of the secreted insulin/IGF antagonist ImpL2. *Dev Cell* **33**, 36-46 (2015).

12. Song, W.*, et al.* Tumor-Derived Ligands Trigger Tumor Growth and Host Wasting via Differential MEK Activation. *Dev Cell* **48**, 277-286.e276 (2019).

13. Newton, H.*, et al.* Systemic muscle wasting and coordinated tumour response drive tumourigenesis. *Nature Communications* **11**, 4653 (2020).

14. Figueroa-Clarevega, A. & Bilder, D. Malignant Drosophila tumors interrupt insulin signaling to induce cachexia-like wasting. *Dev Cell* **33**, 47-55 (2015).

15. Kim, J.*, et al.* Tumor-induced disruption of the blood-brain barrier promotes host death. *Dev Cell* **56**, 2712-2721.e2714 (2021).
